# Short-term exposure to filter-bubble recommendation systems has limited polarization effects: Naturalistic experiments on YouTube

**DOI:** 10.1073/pnas.2318127122

**Published:** 2025-02-18

**Authors:** Naijia Liu, Xinlan Emily Hu, Yasemin Savas, Matthew A. Baum, Adam J. Berinsky, Allison J. B. Chaney, Christopher Lucas, Rei Mariman, Justin de Benedictis-Kessner, Andrew M. Guess, Dean Knox, Brandon M. Stewart

**Affiliations:** ^a^Department of Government, Harvard University, Cambridge, MA 02138; ^b^Operations, Information, Decisions Department, the Wharton School, University of Pennsylvania, Philadelphia, PA 19104; ^c^Department of Sociology, Princeton University, Princeton, NJ 08544; ^d^John F. Kennedy School of Government, Harvard University, Cambridge, MA 02138; ^e^Department of Political Science, Massachusetts Institute of Technology, Cambridge, MA 02142; ^f^Department of Marketing, Fuqua School of Business, Duke University, Durham, NC 27708; ^g^Department of Political Science, Washington University in St. Louis, St. Louis, MO 63130; ^h^Department of Politics and School of Public and International Affairs, Princeton University, Princeton, NJ 08544; ^i^Department of Sociology and Office of Population Research, Princeton University, Princeton, NJ 08544

**Keywords:** political polarization, recommendation systems, experiment

## Abstract

An enormous body of literature argues that recommendation algorithms drive political polarization by creating “filter bubbles” and “rabbit holes.” Using four experiments with nearly 9,000 participants, we show that manipulating algorithmic recommendations to create these conditions has limited effects on opinions. Our experiments employ a custom-built video platform with a naturalistic, YouTube-like interface presenting real YouTube videos and recommendations. We experimentally manipulate YouTube’s actual recommendation algorithm to simulate filter bubbles and rabbit holes by presenting ideologically balanced and slanted choices. Our design allows us to intervene in a feedback loop that has confounded the study of algorithmic polarization—the complex interplay between supply of recommendations and user demand for content—to examine downstream effects on policy attitudes. We use over 130,000 experimentally manipulated recommendations and 31,000 platform interactions to estimate how recommendation algorithms alter users’ media consumption decisions and, indirectly, their political attitudes. Our results cast doubt on widely circulating theories of algorithmic polarization by showing that even heavy-handed (although short-term) perturbations of real-world recommendations have limited causal effects on policy attitudes. Given our inability to detect consistent evidence for algorithmic effects, we argue the burden of proof for claims about algorithm-induced polarization has shifted. Our methodology, which captures and modifies the output of real-world recommendation algorithms, offers a path forward for future investigations of black-box artificial intelligence systems. Our findings reveal practical limits to effect sizes that are feasibly detectable in academic experiments.

## Significance

Using an experimental design that mimics the YouTube interface, we demonstrate that presenting people with more partisan video recommendations has no detectable polarizing effects on users’ attitudes in the short term. We conduct four experiments on two different political issues including just under 9,000 users. In the design, we allow users to watch videos on a YouTube-like platform and choose videos from a set of experimentally manipulated recommendations. While we cannot rule out effects from long-term exposure or to small vulnerable subsets of users, our evidence is not consistent with prevailing popular narratives about YouTube recommendation systems radicalizing users en masse

The ubiquity of online media consumption has led to concern about partisan “information bubbles” that are thought to increasingly contribute to an underinformed and polarized public (1). Prior work has focused on cable TV or textual news, but with the rise of new forms of media, the most pressing questions concern online video platforms where content is discovered through algorithmic recommendations. Critics argue that platforms such as YouTube could be polarizing their users in unprecedented ways (2). The ramifications are immense: More than 2.1 billion users log in to YouTube monthly, and popular political extremists broadcast to tens of millions of subscribers.

Empirical research in this setting has long been stymied by enduring challenges in the causal analysis of media consumption and its effects. While observational studies allow researchers to study media in realistic settings, they often conflate the content’s persuasiveness with selective consumption by those who already believe its message. Experiments mitigate the issue of self-selection by randomly assigning participants to view specific videos, but this comes at a cost: Forced assignment often eliminates freedom of consumption or limits choices in ways that do not reflect real-world settings (3, 4). In turn, this makes experimental results difficult to generalize to the real-world challenges of greatest importance—whether media causes polarization among the people who choose to consume it. In our context, ideological polarization, or radicalization, means a shift in opinions toward the relative extremes along a continuum of opinion about a specific political issue (5). The challenges of studying this phenomenon are heightened for social-media platforms—such as YouTube, Facebook, X (Twitter), or TikTok—because their underlying recommendation algorithms are black boxes the inner workings of which academic researchers cannot directly observe. While work such as www.their.tube has powerfully demonstrated that recommendation systems can in theory supply politically polarized recommendations, evidence on the prevalence of this polarized supply has been limited. More importantly, few existing research designs attempt to connect this algorithm-induced supply of polarized media to demand-side changes in consumer watching decisions, much less the effects of this consumption in terms of polarized attitudes and behavior. The result is a contradictory set of findings providing differing estimates of the amount of potentially polarizing content, but few investigations of the effects of that content (6–13).

To test widely circulating theories about this phenomenon, we develop an experimental platform and design to estimate the causal effects of black-box recommendation systems on media consumption, attitudes, and behavior. We designed and built an online video interface that resembles YouTube and allows users to navigate a realistic network of recommendations—the set of options shown after an initial “seed” video, the subsequent options that follow after the chosen second video, and so on—that are directly scraped from the existing YouTube algorithm. Starting with this naturalistic reproduction, which maximizes the ecological validity of the study, we randomly perturb the recommendations shown to users after each video. We continuously track demand-side behaviors such as choices among the recommended videos, skipping decisions, likes, dislikes, and “save to watchlist” actions during their 15 to 30-min watch session.

Existing theories of polarizing recommendations come in two variations: “filter bubbles,” which serve recommendations that are similar to previously consumed content (14), and “rabbit holes” which offer increasingly extreme content over time (2). We address both of these phenomena in separate experiments—focusing on filter bubbles which we find to be more empirically common on YouTube.

**Fig. 1.**
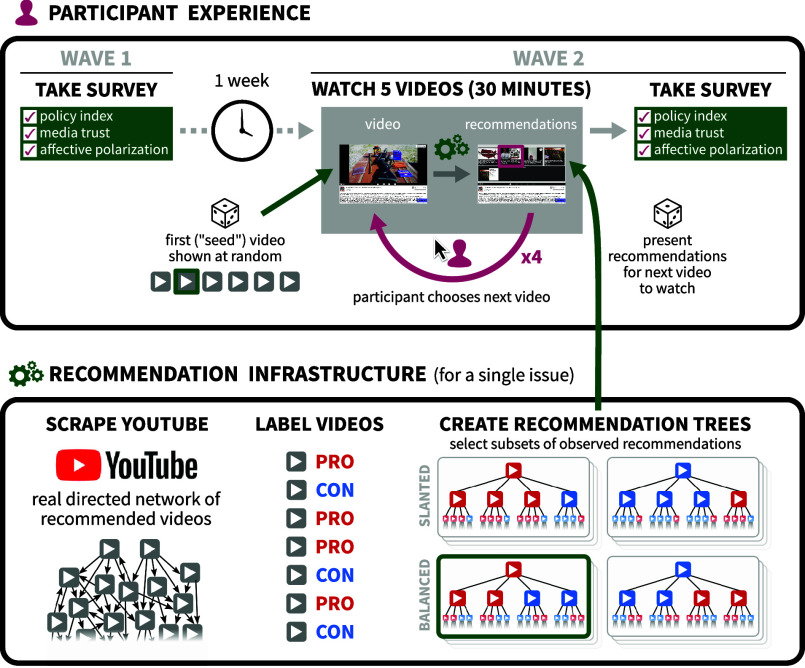
An overview of the design in Studies 1 to 3. In the first wave, participants answer a series of questions. One week later in the second wave, participants are randomized to a seed video and a recommendation system from which they choose future videos to watch. After watching five videos, they take a follow-up survey. Study 4 uses a similar design in one wave but participants are randomly assigned to a sequence of either constant or increasingly extreme content.

In the filter bubble experiments (Studies 1 to 3) we use a multiwave survey to explore how experimental intervention causes individuals to change policy opinions, increase partisan animosity, or alter attitudes toward mainstream media in two issue areas. Fig. 1 provides a graphical overview of the design. We then evaluate the rabbit hole hypothesis by constructing curated sets of video sequences that are either constant or increasing in extremity and randomly assign participants to watch them in a single-wave study. Below, we present the results of these four studies with a combined N of 8,883. Our analyses draw on over 130,000 experimentally manipulated supply-side video recommendations; more than 31,000 demand-side user decisions to watch, like, dislike, and save to watchlists; and a host of outcomes that measure recommendation-system effects on affective polarization, media trust, and policy attitudes. All experiments were preregistered with the Open Science Framework (see *SI Appendix*, section 3).

We consistently find that while changes in the recommendation algorithm do affect user demand by shifting the types of videos consumed and the amount of time spent on the platform, they ultimately did not produce the theorized effects on political attitudes in a substantial way. This is despite the fact that we do see effects from the assignment of initial seed videos to ideological moderates. We emphasize that this evidence does not rule out the possibility that YouTube is a radicalizing force in American politics because our design does not address long-term exposure or potential effects in particularly susceptible subpopulations. Our study also captures effects only at a particular moment in time—it remains possible that earlier versions of YouTube’s recommendation system had radicalizing effects that were addressed in response to criticism. Yet, in the most credible study of algorithmic polarization to date, we observe only minimal attitudinal shifts as a result of more extreme recommendations, calling into question widely circulating, unequivocal claims about their influence on political polarization. We are not claiming that polarization from recommendation systems cannot be found anywhere, but the consistent lack of short-term effects suggests that it is not everywhere.

In the next section, we briefly review the related literature and describe the testable implications of existing theories that characterize YouTube as a radicalizing system, both in terms of shifts in user demand and the effects of those shifts on political attitudes. In Section 2, we describe our survey experimental design, the video-recommendation platform that we built to conduct it, and a manipulation check we conducted to evaluate whether users perceive partisan signals in thumbnails. In Section 3, we present the results from four studies on two policy issues—gun control and minimum wage—detailing the lack of evidence for claims about algorithmic polarization. In the final section, we place these findings in a broader context—re-emphasizing the limitations of what we can know about long-term effects or small, but vulnerable, populations—and propose directions for future work.

## 1. Radicalizing Potential of Recommendation Systems

One of the primary theoretical perspectives on YouTube—and algorithmic recommendation systems more generally—contends that users’ initial preferences trigger algorithmic personalization, which can generate polarization ( 2, see e.g., ref.][). Recommendation algorithms maximize certain outcomes (watch time, engagement) at the expense of others (long-term satisfaction, information quality). However, the inner workings of these systems are generally opaque apart from occasional published technical details (15–17). Prior work has noted that the circular logic of recommendation-system development, which trains recommendation algorithms on user data that is itself driven by prior algorithmic recommendations, can lead to unanticipated consequences such as homogenization of user behavior (18).

### 1.1. Theories of Polarization.

We draw a distinction between two forms of the argument that recommendation systems contribute to polarization. One is that filter bubbles can form when ranking systems are optimized for predicted engagement, resulting in potentially polarizing effects of consuming information from the resulting like-minded sources that appear on the feed (1, 14). Research on the filter-bubble effect has often focused on personalized search results on specific queries (19), with recent studies finding a strong role for user preferences and heterogeneity across topics (20). Looking specifically at social media, the most recent evidence shows that content from congenial or “like-minded” sources constitute a significant share of what users see on Facebook (21), and this is driven in part (though not mostly) by algorithmic personalization (22).

The second form of the argument leverages the rabbit hole concept which posits a sequential element to algorithmic curation. In contrast to filter bubbles—which only suggest algorithmic curation will provide users with more ideologically congenial content, compared to an uncurated platform—rabbit holes additionally imply that the curation process serves up content from one’s preferred side but with increasing extremity or intensity over time. For example, Tufekci (2) argues that YouTube’s recommendation algorithm “promotes, recommends and disseminates videos in a manner that appears to constantly up the stakes.” She suggests that this occurs via a feedback mechanism in which algorithmic curation reinforces users’ preferences, which then drive even more extreme content over time. Similarly, a Wall Street Journal feature on YouTube recommendations found that “[w]hen users show a political bias in what they choose to view, YouTube typically recommends videos that echo those biases, often with more-extreme viewpoints” (23). Similar arguments have been made in a diverse academic literature that—based on a mix of informal reasoning, theoretical models, and observational studies—argued that the supply of slanted content, both by itself and through its interaction with user demand, has adverse effects on political attitudes (14, 24–28). Experimental evidence on the subject has been far more mixed. Though there has been some evidence of harmful polarization effects (29–31), other well-powered experiments have produced null findings (21, 32) or even suggested benefits from algorithms that shield people from opposing viewpoints that would provoke backlash (33). A growing number of studies, however, simply take the conventional wisdom about algorithmic harms for granted, using these concerns to motivate the study of indirectly related questions about the supply of slanted content (10, 34–36), user demand for it (20, 37, 38), or both (8, 9, 39, 40).

To show the existence of rabbit hole phenomena, two elements must be established: 1) user preferences must lead to algorithmic curation of congenial videos or channels, and 2) algorithmically served videos must become more extreme over time. Many existing studies of YouTube recommendations focus on the first element. For example, Hosseinmardi et al. (9) find a correlation between preferences for content elsewhere on the internet and political video channels on YouTube. Other studies attempt to estimate “pathways” between categories of content on YouTube, such as channels classified as mainstream or radical (7, 8). We are aware of one study that attempts to estimate whether algorithmically driven video consumption becomes more extreme over time: Haroon et al. (41) show a significant—but slight—increase in the average extremity of videos shown to sock-puppet accounts as more up-next recommendations are followed. However, extremity in this study was determined by estimating the ideology of Twitter accounts that share links to specific videos, a method that may be sensitive to the sparsity of the data.

### 1.2. Approaches to Studying Opinion Change.

The circular interaction between past preferences (which shape the set of recommended videos and how users choose among them) and consumption (which shapes future preferences by changing recommendations and user tastes) leads to severe challenges in the study of media persuasion and preference formation. There is a venerable social-science tradition that has used experiments to understand the persuasive effects of films and videos (42). The standard “forced-choice” design assigns one group to a video condition with another assigned to a control or placebo condition, with neither group provided alternatives or given the option to avoid the stimulus ( 43, e.g., ref.][). This allows analysts to cleanly estimate the effect of forcing the entire population to consume one piece of media instead of another. Yet this counterfactual quantity focuses entirely on media supply and neglects the interplay with user demand. As a result, it is of limited value in studying high-choice environments when self-selection is the primary determinant of media selection. More recently, scholars have studied the interaction of user choice and media effects in related literature on partisan cable news (3, 4, 44). A key insight of these works is that the persuasiveness of partisan news varies across individuals with different preferences: Effects are different for those who prefer entertainment, compared to those who prefer ideologically congenial news sources (45). Related insights inform the current literature on the effects of digital media and social media (31–33).

To account for the role of user demand in persuasion, Arceneaux et al. (3) develop active audience theory, which emphasizes people’s goals and conscious habits in deciding what types of content to consume. On the one hand, some people may prefer to consume partisan or biased media (44, 46, 47); on the other, this media diet can alter future preferences. Crucially, the interaction of these phenomena could unleash a spiral of rising polarization and self-isolation (48). Recent work has sought to estimate the causal effect of partisan media specifically on those who choose to consume it (3, 49, 50)—the quantity that matters most in real-world polarization, since much of the population voluntarily opts out of exposure.

The existing literature on algorithmic recommendations can similarly be broken down in terms of media recommendations (supply), media consumption (user demand), and the effects of this consumption on user preferences and attitudes. Existing work has generally focused on understanding the demand side of the problem. In an influential study, Ribeiro et al. (8) collect video metadata, comments, and recommendations covering 349 channels, more than 330,000 videos, and nearly 6 million commenting users. By connecting commenters across videos and following networks of recommendations, the authors find that commenters in less-extreme “alt-lite” and “intellectual dark web” (IDW) channels are more likely to subsequently comment on more extreme “alt-right” channels. They also observe a substantial share of channel recommendations from alt-lite and IDW videos to alt-right channels, but they find no evidence of direct recommendations from mainstream media to alt-right channels. These findings are consistent with alternative but less extreme sources serving as a “gateway” to more extremist content—but this observational audit methodology cannot disentangle the role of the algorithm from that of user preferences, nor can it assess the effect of consumption on attitudes or behavior. Brown et al. (10) use a different design to examine the correlation between the supply of algorithmic recommendations and policy attitudes at a particular moment in time, breaking into the supply–demand loop by eliminating the role of user choice. Participants log into their own accounts and are then given a starting “seed” video as well as instructions to click on the first, second, etc. video recommendation. The network of recommendations is then explored to a depth of over 20 choices. They estimate a modest correlation between self-reported ideology and the average slant of recommended videos but, counterintuitively, find a consistent center-right bias in the ideological slant of recommended videos for all users. Haroon et al. (12) extend this approach to examine the interaction between supply and demand, using 100,000 automated “sock-puppet” accounts to simulate user behavior; they argue that YouTube’s recommendation algorithm direct right-wing users to ideologically extreme content. However, in another experiment using sock-puppet accounts that initially mimic the browsing history of real users, Hosseinmardi et al. (13) show that YouTube’s recommendations quickly “forgets” a user’s prior extremist history if they switch back to moderate content. Haroon et al. (41) show through a sock-puppet study and a longitudinal experiment on 2,000+ frequent YouTube users that nudges can increase consumption of balanced news and minimize ideological imbalance, but that there are no detectable effects on attitudes.

Other work has used observational methods to study the correlation between demand and policy attitudes, rather than seeking to estimate how an intervention would change those attitudes. Hosseinmardi et al. (9) examine the broader media ecosystem by tracking web-browsing behavior from a large representative sample; they show that video views often arise from external links on other sites, rather than the recommendation system itself, and conclude that consumption of radical content is related to both on- and off-platform content preferences. Chen et. al. (11) similarly combine a national sample and browser plugins to show that consumption of alternative and extreme content, though relatively rare, is associated with attitudes of hostile sexism; they further show that viewers tend to be subscribed to channels that deliver this content. This suggests that personal attitudes and preferences—as reflected in the decision to subscribe to a channel—are important factors driving consumption of extremist content, though it does not rule out the possibility that algorithmic recommendation systems play a role in initially exposing viewers to this content.

Taken together, the results imply that though algorithmic recommendations may shape the experience of using video platforms, their effects may be subtler and more complex than we might expect from a simple rabbit hole model of radicalization. At a minimum, observational evidence suggests that users’ choices to consume content can also reflect their preexisting attitudes and nonplatform preferences. There is also limited evidence that rabbit holes exist in practice. While much of the work has focused on the recommendation or consumption of ideological content, there is very little research on the causal persuasive effects of the self-selected content or the algorithms that recommend it.

### 1.3. Testable Implications.

We build on these existing lines of work by developing a realistic experiment to estimate how changes in recommendation-system design (a supply-side intervention) affect user interactions with the platform (demand for content) and, through changes in the content consumed, ultimately cause changes in political attitudes. In our main design, participants are presented with an initial “seed” video and, after choosing to watch or skip it, are offered four videos to select for the next round. By carefully pruning and rewiring the real-world YouTube recommendation network, we create two realistic recommendation algorithms: a “slanted” algorithm (which we call 3/1) that primarily gives options from the same ideological perspective as the most recently watched video (mirroring a filter bubble) and a “balanced” algorithm (which we call 2/2) that presents an equal mix of supporting and opposing perspectives. Unlike existing work on the persuasive effects of partisan media, we allow users to choose up to five videos in a single, continuous viewing session. This design mimics real-world viewing behavior and allows us to account for how demand-side choices shape the supply of videos subsequently available to view in a sequence. By experimentally manipulating actual YouTube recommendation networks, our approach combines the causal identification of recent media-persuasion experimental research with the realism of recommendation-system audit research. This produces a research design that can credibly estimate the causal persuasive effects of recommendation algorithms. It allows users to choose the content that they wish to consume, but it prevents this freedom of choice from confounding inferences about the algorithm’s downstream effects. By increasing the slant of the algorithm beyond the current levels, we also side-step a challenge inherent in observational studies conducted after YouTube’s 2019 algorithm updates—the fact that they are limited in what they can say about algorithm’s polarizing potential before those changes were made (11). Platforms like YouTube are a moving target (51, 52) but our design suggests that even implementing a dramatically more slanted algorithm has limited effects on opinion formation.

In the analyses that follow, we argue that widely circulating claims about algorithmic polarization imply four testable hypotheses. First, because user behavior is heavily shaped by platform affordances and recommendation systems are designed to influence video consumption, prior observational work (8) suggests that random assignment to a balanced or slanted algorithm will powerfully affect user demand, as measured by the content that users immediately choose to consume. Second, since online video systems are part of a broader alternative-media ecosystem (53), supply-side changes in the recommended content may affect other, second-order components of demand, such as the trust they place in various types of news sources (32, 54, 55). This builds on previous work that found one-sided media consumption drives distrust of the news media more generally (54–56). One-sided media consumption can eventually lead to more worrisome outcomes, such as reduced reliance on new information and lowered opinions of out-party politicians (44).

Because slanted videos are believed to have a persuasive effect, a third testable hypothesis is that randomized assignment to different algorithms will indirectly cause changes in users’ specific attitudes on the topic of the videos—in our studies, gun control or minimum wage. Such effects could unfold through a variety of mechanisms, including framing of the issue (57), cue-taking (58), or new policy-relevant facts (59). Finally, we examine whether manipulating the recommendation algorithm has a more general second-order impact on affective polarization, rather than just issue-specific polarization. This is because prior work has shown traditional media’s role in affective polarization (60)—emotional attachments to one’s partisan ingroup, as well as distaste for the outgroup—which may be heightened by the slanted and inflammatory content that recommendation systems often suggest.

While existing claims imply these four testable hypotheses, a pressing claim is whether we would expect those effects to appear in a short-exposure experiment. We describe our study as short-exposure because it is not positioned to identify effects that might come from prolonged exposure of watching videos over many months or years. Aside from innovative encouragement experiments (33) which encourage, but do not force, participants to consume media outside of real-world settings, the majority of the experimental literature is based on short exposures. We conducted an expansive review of all PNAS studies in the last decade that met two criteria: They 1) presented a treatment (e.g., video clips, reading materials, or images) in a human-subjects experiment and 2) examined participants’ decisions and opinions following the intervention (see *SI Appendix*, section 18 for details). The median length of exposure to persuasion stimuli was 101 s. Many of these studies deliver quite strong effects such as Tappin et al. (61), which examined the persuasiveness of microtargeted videos on policy attitudes. The average duration of their video stimuli was 52 s and their maximum exposure was 70 s. Like many other studies with short exposure to media stimuli, they demonstrate that these interventions can indeed have significant effects on deeply entrenched political attitudes (they studied immigration and welfare policy, which we view as roughly comparable to our gun-rights issue and far more entrenched than our minimum-wage issue). At an average of 23 min, our “short” exposure is an order of magnitude longer than these prior studies, providing a credible empirical evaluation of the first-order implications of the existing narrative on algorithmic polarization.

## 2. Experimental Design

**Fig. 2.**
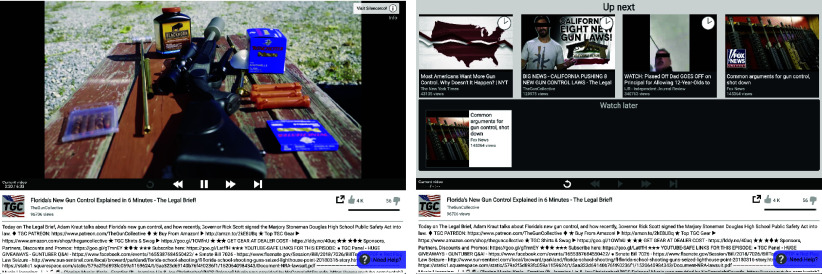
Video platform interface and recommendations. The *Left* panel shows the video-watching interface for an example video in Study 1, and the *Right* panel shows an example of recommendations that were presented to respondents after the video.

To address challenges posed by supply–demand interplay, we developed an experimental design that randomly manipulates video recommendations through a custom-built, YouTube-like platform (Fig. 2). We provide brief details below, deferring additional details to *Materials and Methods*.

We gathered real YouTube videos on two policy issues (more on the issues below), collected actual YouTube recommendations for these videos, experimentally manipulated these recommendations to be slanted or balanced, and then sequentially presented the videos and their following recommendations to experimental subjects in a realistic choice environment. We continuously monitored how users chose among recommended videos, whether they skipped forward or watched videos in their entirety, and how they otherwise positively or negatively interacted with the video. To test whether recommendation algorithms had an effect on attitudes, subjects were surveyed in two waves occurring roughly one week before and immediately after using the video platform.^*^

Our platform and its recommendations were designed to closely approximate both the viewing experience and the algorithmic recommendations of YouTube. Upon entrance to the platform, respondents were shown a “seed” video on a topical policy issue: on gun control in Study 1 or on the minimum wage in Studies 2 and 3. At the conclusion of the video, respondents were presented with four recommended videos to watch next, drawn from the actual YouTube recommendation network. Respondents selected another video from the recommendations, watched that video, and then were presented with another set of recommendations. Each respondent watched up to five videos, with four opportunities to choose among different sets of recommendations. Respondents were required to watch at least 30 s of each video before they were allowed to skip ahead to the end of the video. Throughout their time on the video platform, respondents could interact with the platform by indicating whether they liked or disliked the video they were watching, and they could save the current or recommended videos to watch later.

Videos on the selected policy topics, along with their recommendations, were identified via the YouTube application programming interface (API), validated, and classified for valence. Our experiments manipulated both the slant of the initial “seed” video (liberal or conservative) and the mix of recommendations presented to subjects after they watched each video (balanced or slanted in the direction of the previous video), for a total of four conditions. Based on the pretreatment political attitudes, respondents were divided into liberal, moderate, and conservative terciles with the ideologues (liberals and conservatives) only being shown the like-minded seed. After watching or skipping each video, respondents were presented with four recommended videos that were either “balanced” (two recommendations matching the ideological direction of the previous videos and two from the opposite perspective) or “slanted” (three matching and one opposing).

Our main outcome, policy attitude, is measured with an index formed from responses to five (Study 1) or eight (Studies 2 to 4) survey questions on the relevant policy, which we averaged into a measure that ranged from 0 (most liberal) to 1 (most conservative). We also include measures of media-trust, behavior on platform (interactions with the video platform) and affective polarization. We analyze posttreatment attitudes using regressions that control for a set of attitudes and demographic characteristics that were measured pretreatment per our preanalysis plan. Our main analyses examine the effect of the slanted recommendation algorithm (vs. the balanced algorithm) on our outcomes.

We recruited large and diverse U.S.-based samples (within the confines of modern survey sampling) across all studies using MTurk via CloudResearch and YouGov (Studies 1 to 3 include approximately 2,500 participants each). Study 1 was started in June 2021, Studies 2 and 3 were started in April 2022, and Study 4 was started in May 2024.

### 2.1. Policy Issues.

In order to have a well-defined measure of video valence, extremity, and policy attitude, we limit our studies to one policy issue each. Study 1 covers gun control and Studies 2 to 4 covers minimum wage. This naturally induces a limitation that we can only speak directly to these topic areas. The claims in the polarization literature have largely not been qualified by topic. Indeed, Tufekci (2) argues that (at least in 2018) YouTube was “radicaliz[ing] billions of people” across countless issue areas—vaccines, diet, nutrition, exercise, gun policy, white supremacy, 9/11, and more. In our choice of issues we had to trade-off between issues raised in the polarization literature but where there would be serious ethical implications (e.g., white nationalism, pro-ISIS videos, and vaccine skepticism) and more common policy topics. We chose gun control because it connects with some of the most visceral examples of rabbit holes (e.g., conspiracies in school shootings). We chose minimum wage to find a case that was high profile, but less divided along partisan lines. In the qualitative case-selection language, the strong and weak partisan divisions on these topics of gun control and minimum wage policy, respectively, mean they could perhaps be regarded as “least likely” and “most likely” issues for persuasion effects (at least among high-profile topics). Regardless, we emphasize that our evidence is specific to the gun control and minimum wage debate; it could be that effects exist in other topic areas, particularly on less-salient issues where opinions may be more movable.

### 2.2. “First Impressions” Experiment.

Our design changes the balance of recommendations and allows users to choose videos in an ecologically valid way—by observing the thumbnail, channel name, and view count. This does not ensure that they are able to select content based on valence if they are not able to perceive the valence from the thumbnail. In an experiment reported in *SI Appendix*, section 13, we use the video recommendation interface to collect participant evaluations of the partisan leaning of a video. Our results show that participants have a higher-than-chance ability of discerning the political leaning of a video based on the recommendation page. However, there is substantial heterogeneity across topics and video ideology, with conservative minimum wage videos being particularly easy to guess and liberal gun control videos being particularly challenging. We also use a computational baseline (GPT-4V) to assess how much visual information is present even if participants do not discern it. We find that GPT-4V is able to achieve 84% accuracy overall (91% for minimum wage and 69% for gun control)—far exceeding human performance.

### 2.3. Rabbit Hole Experiment (Study 4).

Studies 1 to 3 take the existing YouTube algorithm as a starting point and artificially “slant” it to boost prevalence of similar ideological position (magnifying the filter bubble phenomenon). Our real-world recommendation data suggest that this captures real-world patterns on YouTube well. We analyzed video transcripts to measure their ideological extremity and found that recommendations did not get increasingly extreme—in fact, we found that extreme videos led to recommendations that were slightly more moderate, a pattern that is consistent with regression to the mean (see *SI Appendix*, section 16). Consequently, the experiments derived from this real-world data also do not get more extreme; in other words, Studies 1 to 3 capture the filter bubble phenomenon but not the rabbit hole. This is consistent with observational work on YouTube using sock-puppets by Haroon et al. (12) who found only “substantively small” extremity increases over video sequences.

For Study 4, we developed an experiment that would artificially intensify the extremity of videos to assess the effects that viewing such sequences might have on minimum wage political opinions. In this experiment, we again divided participants into three groups (conservatives, liberals, and moderates). Conservatives and liberals were assigned to an ideologically aligned sequence that was constructed to be either constant in extremity or increasing. Moderates were assigned one of the four types of sequences. In contrast with Studies 1 to 3, this study was conducted entirely in one wave (asking opinions before and after the video viewing) and did not involve choosing videos to view. In this sense, it provided a substantially stronger, but less ecologically valid, treatment.

## 3. Results

We first present side-by-side results from Studies 1 to 3 to permit comparisons across issue areas and sampling frames. Our first two sets of results examine the algorithmic effect of being assigned to an ideologically slanted recommendation system, compared to a balanced one (corresponding theoretically to a “filter bubble effect”). We begin with algorithmic effects on liberal and conservative “ideologue” respondents in Section 3.1 before proceeding to algorithmic effects on “moderate” respondents in Section 3.2. In Section 3.3, we present a second set of results that examine the effect of assigning moderate respondents to a liberal seed video, compared to a conservative one, when users are subsequently allowed to freely navigate the recommendation system. As noted above, we fail to find consistent evidence of algorithmic effects, despite calculations for minimum detectable effects (MDEs) that indicate that Studies 1 to 3 were powered to reliably detect algorithmic effects on unit-scale policy attitudes of 0.02 to 0.04 (depending on the study; MDEs are based on conventional 0.05 significance and 80% power cutoffs after accounting for multiple-testing corrections). These MDEs reflect effects that were a priori quite plausible in our experimental setting—indeed, in each of these studies, we observe seed-effect point estimates that are double or even triple the size of the corresponding algorithmic MDE. To address concerns that null effects are due to the filter bubble nature of our algorithmic manipulations in Studies 1 to 3, in Section 3.4, we present the results with a rabbit hole design in Study 4.

Each section below presents estimated effects across a variety of outcome measures. We group these outcomes into four families, based on the hypotheses described in Section 1: 1) demand-side outcomes relating to media consumption and user interaction with the platform; 2) demand-side outcomes about trust in media; 3) attitudinal outcomes measuring issue-specific polarization; and 4) attitudinal outcomes relating to general affective polarization. Throughout, all hypothesis tests reflect multiple-testing corrections as described in *Materials and Methods*. Plots show 90% and 95% CIs with robust SEs; we use color to denote the results of hypothesis testing and emphasize that readers should only interpret results that remain significant after multiple-testing correction.

### 3.1. Algorithmic Effects Among Ideologue Respondents.

**Fig. 3.**
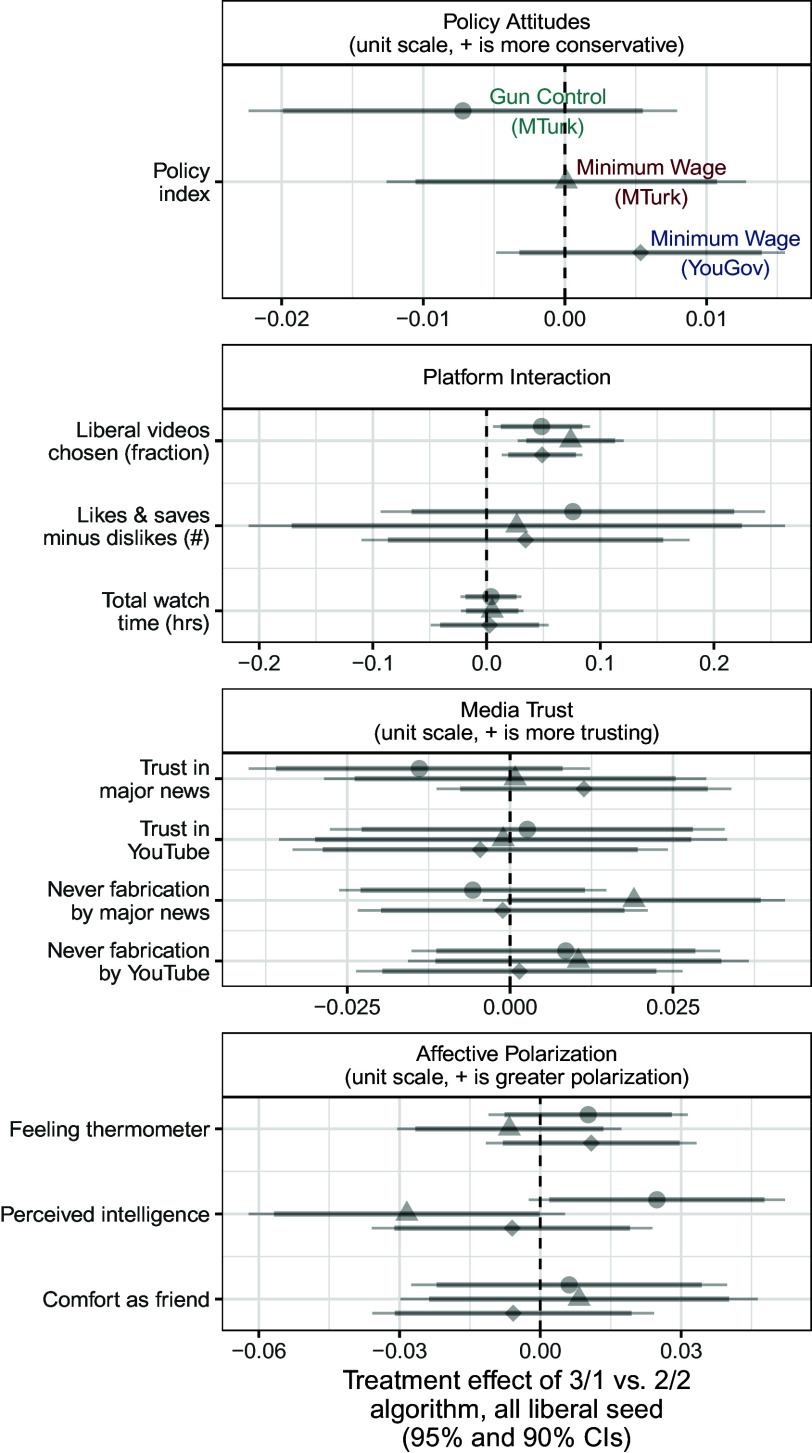
Effects of recommendation algorithm among liberal ideologues. Displays the effects of more algorithmic recommendation slant (vs. balance) on behaviors and attitudes among liberal ideologues (those in the first tercile of pretreatment policy attitudes). Gray points and error bars represent estimated effects that are not statistically significant after implementing multiple testing corrections, while points and error bars in color represent those effects that are still statistically significant after multiple testing corrections.

**Fig. 4.**
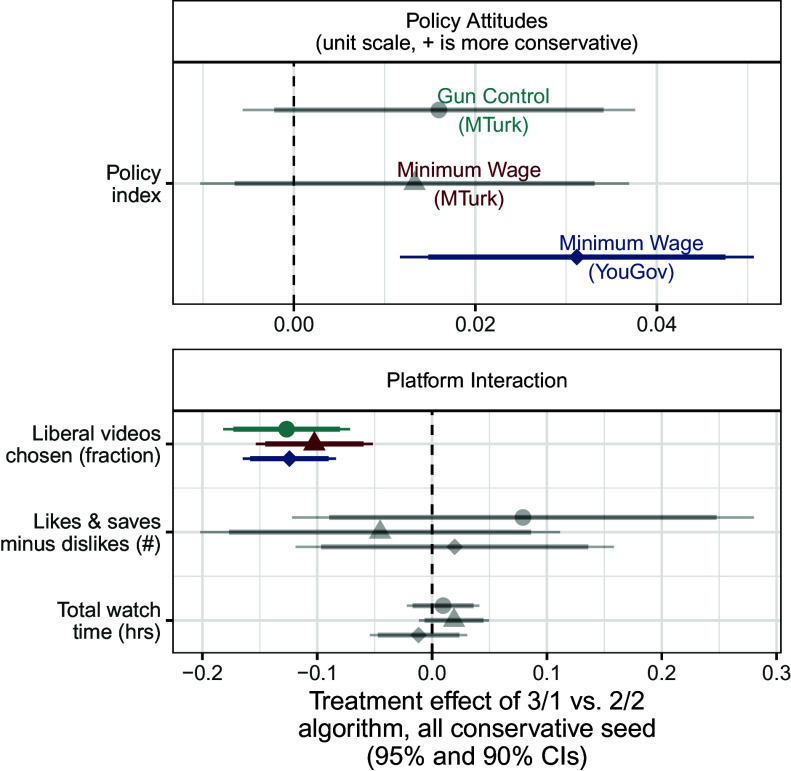
Effects of recommendation algorithm among conservative ideologues. Displays the effects of more algorithmic recommendation slant (vs. balance) on behaviors and attitudes among conservative ideologues (those in the third tercile of pretreatment policy attitudes). Gray points and error bars represent estimated effects that are not statistically significant after implementing multiple testing corrections, while points and error bars in color represent those effects that are still statistically significant after multiple testing corrections. Results on media trust and affective polarization are truncated in Figs. 4–8 but included in multiple testing correction; see complete results in *SI Appendix*, section 10.

We first examine these algorithm-driven effects among ideologues (i.e. those in the lowest and highest terciles of pretreatment policy attitudes). Fig. 3 shows the effects of a more extreme recommendation system among liberal respondents and Fig. 4 shows the same effects among conservative respondents. Each symbol denotes one of our three studies: Filled (turquoise when significant after multiple testing corrections) circles are estimates from our first study, on gun policy; (red) triangles are estimates from the second study, on minimum wage policy with a Mechanical Turk sample; and (blue) diamonds are estimates from our third study on minimum wage policy with a YouGov sample.

The *Top* panel in both sets of results shows the effects on respondents’ policy attitudes. We find few significant effects on these attitudes among ideologues. The one exception is the effect in Study 3 among conservatives. In this study, respondents assigned to view more slanted recommendation videos reported posttreatment attitudes that were slightly more conservative (0.03 units on a 0 to 1 policy index) than respondents assigned to view balanced recommendation videos. Importantly, the estimated effects are quite small. For instance, the upper limit of this 95% CI for the effect of the recommendation system on conservative respondents in Study 1 is 0.04 units on this 0 to 1 policy index, equivalent to 16% of the respondents moving one level up on each of the index’s five-point components.^†^

The *Lower* panel of Fig. 3 shows the effects of the recommendation slant on platform interactions, media trust, and affective polarization (starting with Fig. 4 we truncate results for space; see full results in *SI Appendix*, section 10). For both sets of respondents, we find that a more extreme recommendation system caused respondents to choose more videos from the same ideological slant as the video they had just watched, relative to a balanced set of recommendation videos. Averaging across the three studies, the liberal fraction of videos chosen by liberal respondents assigned to the slanted (3/1) algorithm was 6 percentage points higher than liberal respondents assigned to the balanced (2/2) algorithm. Similarly, the liberal fraction of videos chosen by conservative respondents assigned to the slanted algorithm was 12 percentage points lower than those receiving balanced recommendations. This is consistent with the increased availability of videos: If respondents were choosing randomly, it would be about 12 percentage points higher in the ideological direction of the seed video (which, by design, was matched to the ideological orientation of liberal and conservative respondents). However, we also found that across all four experimental arms—liberal and conservative respondents assigned to balanced and slanted algorithms—respondents watched a significantly larger share of videos supporting their own ideological viewpoint than would be expected under the null hypothesis of random video selection (4.1 to 5.3 p.p. higher depending on arm; all P<0.001). See *SI Appendix*, Section 17.A for details.

### 3.2. Algorithmic Effects Among Moderate Respondents.

**Fig. 5.**
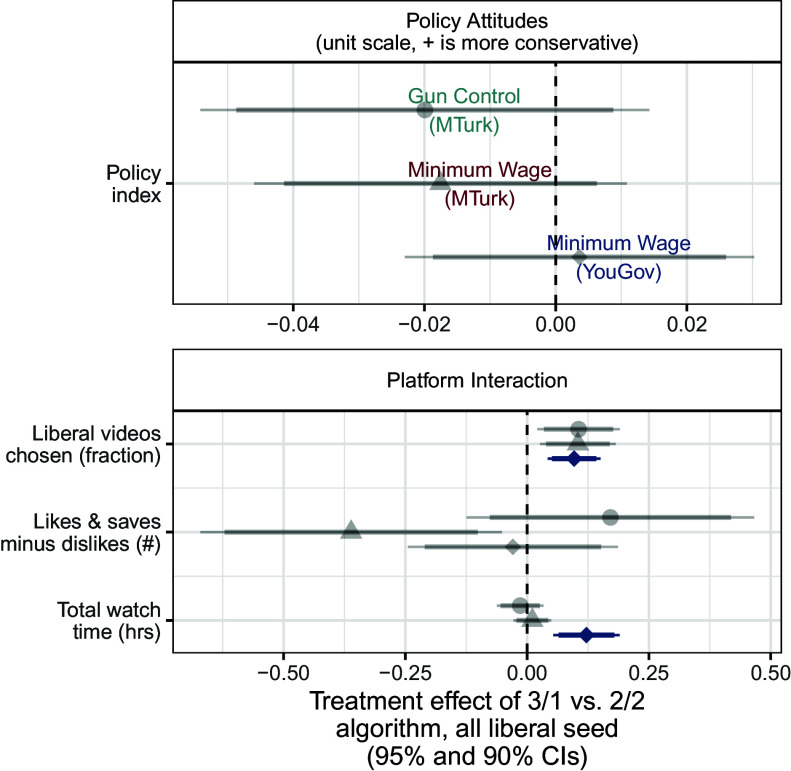
Effects of recommendation algorithm among moderates assigned liberal seed video. The effects of more algorithmic recommendation extremity (vs. balance) on behaviors and attitudes among moderates (those in the middle tercile of pretreatment policy attitudes) assigned to a liberal (i.e., pro-gun control or pro-minimum wage) seed video. Gray points and error bars represent estimated effects that are not statistically significant after implementing multiple testing corrections, while points and error bars in color represent those effects that are still statistically significant after multiple testing corrections.

**Fig. 6.**
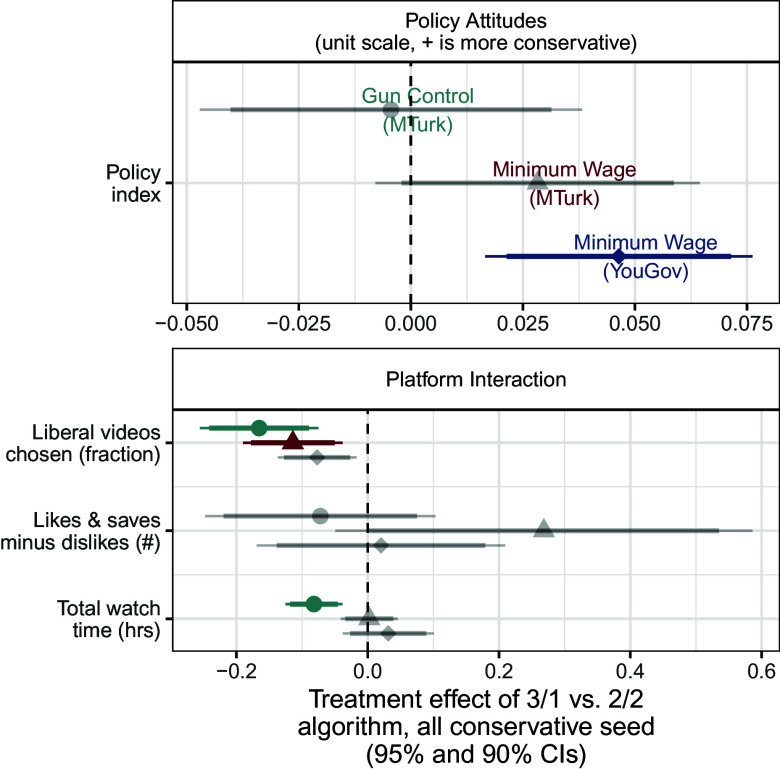
Effects of recommendation algorithm among moderates assigned conservative seed video. The effects of more algorithmic recommendation extremity (vs. balance) on behaviors and attitudes among moderates (those in the middle tercile of pretreatment policy attitudes) assigned to a conservative (i.e., anti-gun control or anti-minimum wage) seed video. Gray points and error bars represent estimated effects that are not statistically significant after implementing multiple testing corrections, while points and error bars in color represent those effects that are still statistically significant after multiple testing corrections.

Our results examining the effects of recommendation algorithms among moderates appear similar. Fig. 5 shows the effect of the more slanted recommendation system for respondents assigned to the liberal seed videos, and Fig. 6 shows the same effect of slanted recommendation system for respondents assigned to the conservative seed videos.

Again, the more slanted (3/1) recommendations appear to influence respondents’ choices of videos, compared to the balanced (2/2) ones, and in two instances significantly affected the amount of time respondents spent on the platform. As in the previous section, respondents assigned to the slanted algorithm chose to watch a higher proportion of videos that resembled the seed video. In other words, respondents assigned to a liberal seed and slanted recommendations were more likely to choose liberal videos, compared to other liberal-seed respondents who received balanced recommendations. Similarly, respondents assigned to a conservative seed and slanted recommendations chose liberal videos at a lower rate, compared to other conservative-seed respondents with balanced recommendations. Among moderates assigned a liberal seed in Study 3, being assigned the slanted recommendations appears to have increased the total time respondents spent on the platform by 7.3 min on average, while moderates assigned a conservative seed video in Study 1 with slanted recommendations appear to have spent 4.9 min less time watching videos on average than those assigned a balanced set of recommendations. These effects are quite large given the average watch time of 23 min. This may be because the sample skews liberal overall, meaning that the “moderate” tercile is still somewhat liberal. In this case, being forced to watch a conservative video and then being presented with three more conservative videos in the first set of recommendations could plausibly decrease satisfaction and time spent on the platform, despite subsequent freedom of choice.

Despite these large effects on media consumption, the slant in recommendations appears to affect political attitudes only minimally among moderates. Nearly all the effects of the recommendation algorithm on policy attitudes, media trust, and affective polarization appear statistically indistinguishable from zero. The one exception is again in Study 3, where it appears that moderate respondents assigned the conservative seed video and slanted recommendations reported opinions that were slightly more conservative (0.05 units on a 0 to 1 scale) than respondents assigned to balanced recommendations. The small size of these estimates and their relatively narrow CIs suggest that the general lack of statistical significance is not simply due to small-sample noise, but rather a genuinely small or nonexistent short-term attitude change. That is, we can rule out anything greater than these quite-modest immediate effects on policy attitudes caused by more extreme recommendation algorithms.

### 3.3. Forced-Exposure Effects Among Moderate Respondents.

**Fig. 7.**
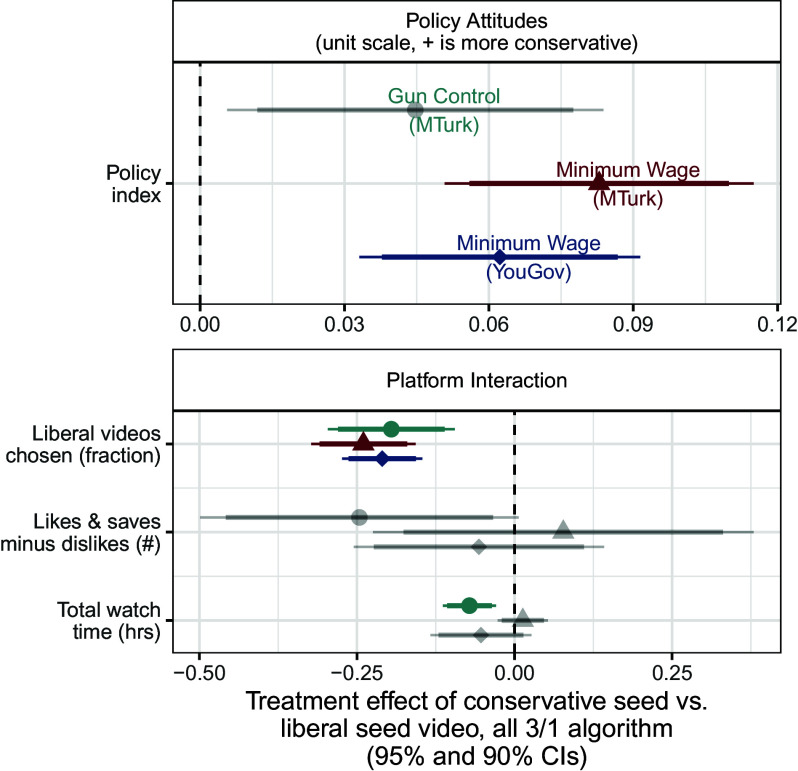
Effects of seed video slant among moderates, 3/1 recommendation algorithm. The effects of a more conservative seed video on behaviors and attitudes among moderates (those in the middle tercile of pretreatment attitudes) assigned to a 3/1 recommendation algorithm. Gray points and error bars represent estimated effects that are not statistically significant after implementing multiple testing corrections, while points and error bars in color represent those effects that are still statistically significant after multiple testing corrections.

**Fig. 8.**
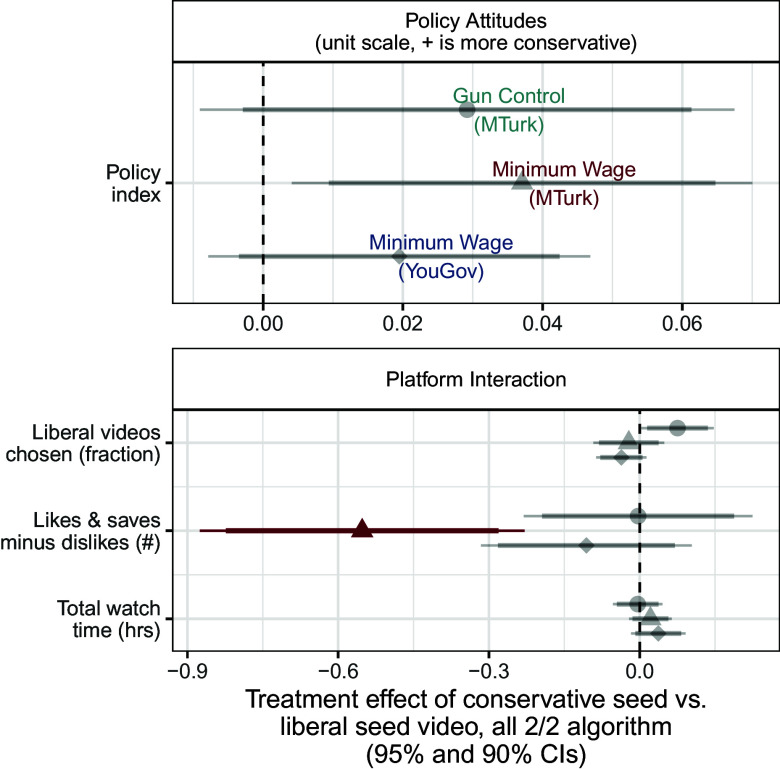
Effects of seed video slant among moderates, 2/2 recommendation algorithm. The effects of a more conservative seed video on behaviors and attitudes among moderates (those in the middle tercile of pretreatment attitudes) assigned to a 2/2 recommendation algorithm. Gray points and error bars represent estimated effects that are not statistically significant after implementing multiple testing corrections, while points and error bars in color represent those effects that are still statistically significant after multiple testing corrections.

We assess the effects of the randomized seed video among moderates. These effects most closely mirror the effects of a traditional randomized forced-exposure study, as they measure the effects of being assigned a conservative rather than a liberal initial video—often referred to as attitudinal persuasion. However, our results differ in that after this forced exposure, we allow users to freely interact with the platform and choose which videos to consume. The results of these analyses are presented in Fig. 7, which shows the difference in outcomes between those respondents assigned to a conservative seed video compared to those assigned to a liberal seed video, among those respondents who received recommendations in a more slanted mix (3/1) and in Fig. 8 among those respondents assigned a more balanced mix (2/2).

The effects of the assigned seed video on moderates’ attitudes, presented in the *Top* panels of Figs. 7 and 8, suggest slight persuasion effects. Respondents assigned to the slanted recommendations who were assigned a conservative seed video reported slightly more conservative policy attitudes than those who were assigned a liberal seed video, as shown in the first panel of Fig. 7. These effects, again, are muted among those respondents who were assigned to the balanced recommendations (Fig. 8). These respondents reported policy attitudes that were not discernibly different when assigned to either the conservative or liberal seed video.

In the slanted recommendation system, being assigned to a conservative video led moderate respondents to choose a much lower fraction of subsequent liberal videos than those assigned to a liberal video, as the second panel in Fig. 7 shows. This effect disappears when moderate respondents are assigned to the balanced recommendations (Fig. 8): Watching a conservative seed video made respondents no more or less likely to choose liberal videos from the recommendations presented to them, as shown in the *Top Right* panel. We observed no other effects on attitudes that were statistically distinguishable from the null hypothesis. That there are some detectable effects on policy from the forced choice assignment gives us confidence that the algorithmic assignment would be able to detect an effect if one existed.

### 3.4. Rabbit Hole Effects (Study 4).

**Fig. 9.**
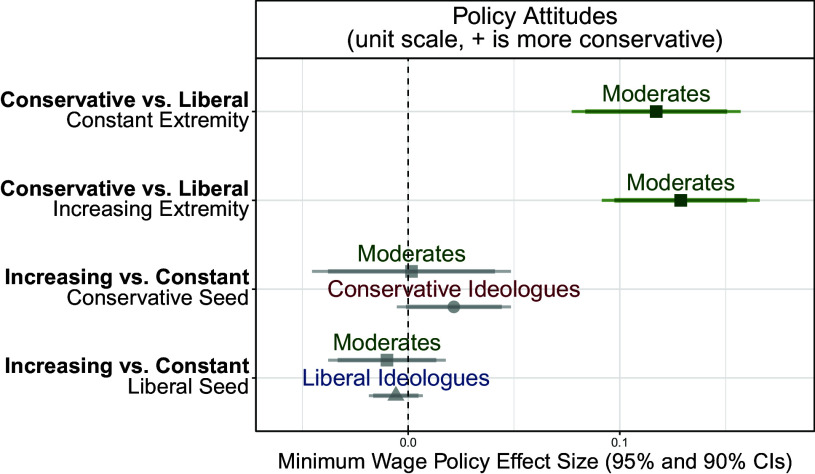
Effects of rabbit hole treatment on policy attitudes. This panel shows the causal contrasts in Study 4. The *Top* two rows show the only significant effects which are the effects of the liberal seed and conservative seed assignment among moderates given that the sequences are constant or increasing. The next two rows show the effects of being assigned to increasing sequences among each group within each assigned seed. Gray points and error bars represent estimated effects that are not statistically significant after implementing multiple testing corrections, while green points and error bars represent those effects that are still statistically significant after multiple testing corrections.

Finally, we present the results of Study 4 which constructed artificial sequences on the minimum wage which were increasing in extremity or held constant in order to test the rabbit hole hypothesis. This design is distinct from Studies 1 to 3 in that it is a single-wave study and respondents are assigned to a fixed sequence of videos (they do not choose recommendations, comparable to the YouTube “Autoplay” experience or the “YouTube Shorts” interface). The results of these analyses are presented in Fig. 9, showing the effects on policy attitudes for different causal contrasts. Assignment to the increasing (vs. constant) sequences appears to have no effect on ideologues or moderates. The only discernible effect is a modest effect of the seed assignment for moderates, consistent with the results in the previous section. This suggests that any algorithmic effect for rabbit holes that exists is likely far smaller than simply watching conservative or liberal video sequences.

Despite these null short-term results on our overall attitudinal index, it remains possible that recommendation algorithms expose viewers to new ways of understanding or interpreting a policy issue that might eventually lead to long-term persuasion. An exploratory reanalysis of Studies 1 to 3 proposed by a reviewer suggested that this might be the case: When extracting more-conceptual survey questions from the overall index to analyze individually, we found patterns that were consistent with algorithmic effects on conservative participants’ understanding of minimum-wage issues. In Study 4, we therefore sought to assess whether algorithmic interventions exposed viewers to unfamiliar information by asking participants about whether they learned anything from watching the videos. Almost 90% of participants reported learning something new. An analysis of the open-ended responses suggests that this learning was diverse including general knowledge, impact on businesses/the economy/poverty, automation, wage stagnation, and political dynamics. We did not find evidence that algorithmic interventions affected the amount of self-reported learning. For details on the exploratory reanalysis of Studies 1 to 3, see *SI Appendix*, section 11; for learning in Study 4, see *SI Appendix*, section 15.

While these findings are useful for contextualizing how rabbit hole systems might operate on YouTube, we emphasize that both our own analysis and (12) suggest that YouTube operates closer to the filter bubble paradigm.

## 4. Discussion and Conclusion

In her 2018 New York Times opinion piece, Zeynep Tufekci provides one of the clearest articulations of YouTube’s role as a radicalizing force in American politics. She paints a picture of YouTube’s ability to recommend users ever more extreme views of what they are already watching—Donald Trump rallies lead to white supremacist rants, Hillary Clinton videos lead to leftist conspiracies, and even jogging leads to ultramarathons. She writes, “It seems as if you are never ‘hard core’ enough for YouTube’s recommendation algorithm. It promotes, recommends and disseminates videos in a manner that appears to constantly up the stakes. Given its billion or so users, YouTube may be one of the most powerful radicalizing instruments of the 21st century (2).”

The implication of this argument—and the assumption of many scientific studies that followed—is not only that YouTube’s recommendation algorithm presents more extreme content to consumers, but that the presentation of this extreme content also changes their opinions and behaviors. This is a worrying claim that applies not only to YouTube but to any of the increasingly numerous online systems that rely on similar recommendation algorithms and, it is claimed, all pose similar potential risks to a democratic society (62). The weaker claim is that recommendation algorithms induce filter bubbles which could produce similar types of opinion changes. Yet if these claims were true, one would imagine that users in our study who were recommended gun-rights videos would have shifted their attitudes substantially toward support of gun rights, and vice versa.

Of course, in many ways, the situation we can test with our experimental design is not the entirety of the story that Tufekci (2) and others describe. It remains possible that months- or years-long exposure to personalized recommendation systems could lead to the conjectured radicalization. Work by Centola (63) has shown that repeated exposure is important to behavior contagions. It also remains possible that there are heterogeneous effects—though we failed to detect heterogeneity in exploratory analyses examining the moderating role of age, political interest, YouTube consumption, and college education.^‡^ We cannot rule out the existence of a small—but highly susceptible—population that cannot be detected with our sample sizes. Finally, it remains possible that the critiques were true at the time that they were written, but that these systems have been subsequently altered.

Nevertheless, by providing real subjects with naturalistic choices over the media they consume, based on actual recommendations from YouTube in nearly 9,000-person randomized controlled trials, our study arguably represents the most credible test of the phenomenon to date. Widespread discussion of YouTube’s radicalizing effects is difficult to reconcile with the fact that we fail to detect consistent evidence of algorithmic polarization in this experiment of either the filter bubble or rabbit hole form. Notably, the narrow CIs on attitudinal effects show that even the maximum effect sizes consistent with our algorithm system results are small, relative to recent experiments on media persuasion with approximately comparable stimuli and our own seed effect estimates. Experiments that allow for respondent choice in videos may tend to have smaller persuasive effects than in traditional forced-choice settings, in part for the simple reason that allowing realistic choice in media consumption leads to fewer users consuming the opposing viewpoints that could persuade them. Our results also align with recent work showing the limits of selective exposure in online media consumption (64, 65), which implies that only a limited set of people will consume highly imbalanced media when given the opportunity. Our results with forced exposure in Study 4 provide larger seed effects, but still no system effects.

Although our study does not provide convincing evidence that the recommendation-system manipulation affected attitudes, we do observe changes in behavior: The balance of recommended videos appears to influence subsequent video selection among moderates and (depending on the seed) total watch time on the platform. Potential decreases in platform watch time as a result of unwanted or unexpected content exemplify the kind of problem that recommendation algorithms are likely intended to solve. This kind of divergence between attitudinal and behavioral effects on social platforms is a potential area for future research. One shortcoming that our study shares with nearly all research on YouTube is that, by taking existing platform recommendations as a starting point, we hold the set of potential videos that could be shown—the supply—as largely fixed, apart from the experimental perturbations in exposure that we induce. Yet like users’ behavior, the production of content is dynamic and subject to incentives. As Munger elaborates (66, 67), the interplay of supply and demand may be an underappreciated factor shaping the choices available to users as they experience the platform, regardless of the specifics of any recommendation system. A full understanding of the impact of streaming video platforms such as YouTube requires simultaneous consideration of interacting and self-reinforcing processes in the supply, demand, and effects of media consumption.

Finally, while our experiments cannot rule out the possibility of some level of radicalization on some subset of the population on YouTube, it provides some guidance on the complexity and scale of an experiment that would be necessary to detect such an effect. Our multiple large-scale survey samples appear to approach the limit of the number of experimental subjects that can currently be recruited for studies as time-intensive as the ones presented here, suggesting that if algorithmic polarization has smaller effects than we were powered to detect, it may be difficult to ever identify them under controlled conditions. Sobering though this conclusion may be, our goal throughout the design and execution of this study has been to maximize our chances of observing a true effect despite hard budgetary constraints. If radicalization were possible, our choice of policy areas—which were selected to vary in their levels of preexisting polarization—should have enabled us to observe attitudinal change. Similarly, our selection of real-world video recommendations from YouTube represents the most realistic attempt that we know of to replicate the slanted recommendation algorithms of social media platforms. The results from our four studies thus collectively suggest that extreme content served by algorithmic recommendation systems has a limited radicalizing influence on political attitudes and behavior, if this influence even exists.

## 5. Materials and Methods

This study has been approved by Princeton University IRB (#12989) and the other institutions via Smart IRB (ID: 3931). All participants consented to the experiment before the initial survey, with consent materials provided in *SI Appendix*, section 1. All replication data and code will be made available in Dataverse on publication.

### 5.1. Collecting the Videos.

We use real recommendations from the YouTube API filtered by topic and stance (see details in *SI Appendix*, section 2). We verify that these correspond with recommendations in actual browser sessions in *SI Appendix*, section 4. As with most prominent audits of the YouTube recommendation algorithm (e.g., refs. 7 and 8), we do not observe personalization based on a user’s watch histories or past engagement. This is an important scope condition, as Haroon et al. (12) find modestly increasingly ideological recommendations for automated sock-puppet accounts. With that said, our experiment targets a well-defined estimand that remains informative for policy questions about algorithmic recommendations, particularly if personalization does not fundamentally change the type of recommendations made (68).

### 5.2. Additional Recruitment and Analysis Details.

Studies 1 and 2 respectively recruited 2,583 and 2,442 respondents on MTurk via CloudResearch (both requiring ≥95% HIT approval rates; Study 1 restricted to workers with ≥100 approved HITs; Study 2 restricted to CloudResearch-approved participants). Study 3 drew 2,826 respondents from YouGov, and Study 4 recruited 1,032 respondents on MTurk via CloudResearch. All studies utilized U.S. participants only. In recruiting our experimental subjects, we used approval requirement qualifications and attempted to recruit a balanced set of political opinions on Mechanical Turk. We had difficulty recruiting respondents that fit these criteria, suggesting that we might be reaching the upper limit of how many people can be recruited on Mechanical Turk for such time-intensive studies. Our sample from a larger and more expensive platform, YouGov, ran into similar issues, suggesting limits to the subject pool available. After exclusion of respondents for repeat taking or zero engagement, the three studies have 1,650, 1,679, and 2,715 respondents respectively in the final analytic sample.

Our main policy attitude outcome is an index formed from responses to five (Study 1) or eight (Studies 2 to 4) survey questions on the relevant policy, which we averaged into a measure that ranged from 0 (most liberal) to 1 (most conservative). Scales were quite reliable, with α of 0.87 to 0.94. We analyzed posttreatment policy, media-trust, and affective-polarization attitudes using regressions that controlled for a pretreatment set of attitudes and demographic characteristics that were measured pretreatment per our preanalysis plan. Platform-interaction outcomes were analyzed similarly, controlling for self-reported YouTube usage and demographic characteristics. To account for the four families of outcomes, we conduct multiple-testing corrections following our preanalysis plan and the recommendations of the literature (69, 70) to control the false discovery rate while properly accounting for the nested nature of the tests. Additional details are available in *SI Appendix*, section 9.

## Supplementary Material

Appendix 01 (PDF)

## Data Availability

Code and experimental data have been deposited in Dataverse at 10.7910/DVN/4WFA5Q (71). Fully replicable code is available on CodeOcean (72).

## Footnotes

^*^Studies 1 and 2 had a third, follow-up survey wave occurring approximately one week after the experimental video-platform session.

^†^Because we find no substantial effects on attitudes in the wave 2 data from studies 1 and 2, we did not analyze the wave 3 data.

^‡^The only significant moderating factor identified in these exploratory analyses was gender.
