# Supplementary material for: Short-term exposure to filter-bubble recommendation systems has limited polarization effects: Naturalistic experiments on YouTube
Source: Proc Natl Acad Sci U S A. 2025 Feb 18;122(8):e2318127122. doi: 10.1073/pnas.2318127122 (PMC11874454; doi:10.1073/pnas.2318127122)
Supplement: Supplementary file 1 — Appendix 01 (PDF) [file pnas.2318127122.sapp.pdf]

PNAS

Supporting Information for

Short-term exposure to "filter-bubble" recommendation systems has limited polarization effects: Naturalistic experiments on YouTube

Naijia Liu, Xinlan Emily Hu, Yasemin Savas, Matthew A. Baum, Adam J. Berinsky, Allison J.B. Chaney, Christopher Lucas, Rei Mariman, Justin de Benedictis-Kessner, Andrew M. Guess, Dean Knox, Brandon M. Stewart

Corresponding Authors: Justin de Benedictis-Kessner, Andrew M. Guess, Dean Knox, and Brandon M. Stewart.
E-mail: jdbk@hks.harvard.edu, aguess@princeton.edu, dcknox@wharton.upenn.edu, bms4@princeton.edu

This PDF file includes:

Supporting text
Figs. S1 to S15
Tables S1 to S11
SI References

Supporting Information Text

1. Consent Materials

A. Wave 1 (Example from Study 2). This research project is being conducted by [AUTHORS]. It is a study to learn more about public opinion on issues in the news. Your participation is voluntary. Participation involves completion of a survey and watching a set of videos. You may choose to not answer any or all questions and to not participate in any portion of the study that you choose. The researchers will not store information that could identify you with your survey responses. Identifying information will not be used in any presentation or publication written about this project. You must be age 18 or older to participate. Questions about this project may be directed to Andrew Guess at [INFORMATION].

If you agree to participate in this survey, click "I agree to participate" below.

B. Wave 2 and Study 4. Your participation in this survey is voluntary. Participation involves completion of a survey and potentially watching a set of videos. You may choose to not answer any or all questions and to not participate in any portion of the study that you choose. The researchers will not store information that could identify you with your survey responses. Identifying information will not be used in any presentation or publication written about this project. You must be age 18 or older to participate. Questions about this project may be directed to [INFORMATION].

If you agree to participate in this survey, click "I agree to participate" below.

C. "First Impressions" Experiment. Participants were instructed on the platform that they could revoke consent by closing their browser and quitting the activity.

2. Creating Recommendation Trees

We base our experiment's recommendations on real recommendations from the YouTube API. To construct our recommendations, we started with "related videos" that the YouTube API identified for each video. From these, we selected the subset of recommendations that were on the same policy topic and took either a liberal or conservative stance on the policy, as determined by a combination of hand coding and supervised machine learning. For both topics, we first conducted a round of coarse regular-expression-based screening for topicality. For gun control, we then used crowd workers on MTurk to create a hand-labeled training set for a cross-validated support vector machine, which was subsequently used to select videos for inclusion. For minimum wage, we used crowdsourcing to classify all videos. Inter-rater agreement ranged from 80% to 85% across multiple rounds of classification. The authors then conducted a final round of manual validation. We arrived at our 3/1 and 2/2 experimental proportions after analyzing YouTube recommendations on the gun-control topic and finding that, among videos with a discernible ideological direction, roughly 60% of recommendations of the same ideology. The 3/1 and 2/2 experimental conditions thus bracket the average real-world proportions, increasing realism.

A. Gun Policy. We collected two starting videos from YouTube about gun policy and used them to construct a recommendation network by querying the YouTube API. We use this directed network to construct recommendation trees representing the different recommendation systems discussed in the paper.

Using the YouTube Data API, we started from two roughly comparable videos (one gun-rights video and one gun-control video), then recursively collected a recommendation network consisting of around 78,000 nodes (unique videos) and 350,000 directed edges (candidate recommendations). The starting videos were selected to ensure that they had a clear stance.[*] Up to 50 non-personalized recommendations were collected for each node, using the `Search` > `relatedToVideoId` functionality.

The videos vary in length from several minutes to several hours; the majority are shorter than 20 minutes.[†] For feasibility of the experiment, we use only videos up to 10 minutes long. We then coarsely screen for topicality by applying a regular-expression filter to their titles.[‡] For videos passing this initial topicality screening, we extracted textual transcripts to classify for ideological valence.

A training set of roughly 2,000 videos was manually labeled as "anti-gun" policy videos, "pro-gun" policy videos, "gun enthusiast" videos, and "other" via workers on Mechanical Turk. A cross-validated (linear) support-vector machine was trained on the training-set transcripts using bag-of-words features, then used to label the full corpus of videos. Regularization determined by cross-validation using the training set. We found that cross-validated SVM attained an accuracy of 82% in unseen test instances of the 2,000 hand-labeled videos. We subset to videos categorized as "anti-gun" or "pro-gun" and subjected the most prominent 283 videos in the network (in terms of the number and position of placements in the recommendation trees described in the next section) to a manual evaluation by authors. Corrections were made as necessary and the trees were regenerated. In the final trees, at least one of the authors had manually reviewed 100% of the seed videos, 93% of the first-level videos, 73% of the second-level videos, 46% of the third-level videos and 30% of the fourth-level videos.

For each of the 10 seed videos, we made 20 trees for each recommendation system condition. When a respondent was randomly assigned to a seed/system combination, we randomly chose one of the 20 unique trees to assign. We continually conducted checks for subsequently deleted videos to remove recommendation trees that contained them.

[*] We used a video from Fox News and a video from *The Atlantic*.

[†] The 25th percentile in video length is 6 minutes, the median is 10 minutes, and the 75th percentile is 17.5 minutes.

[‡] The filter was hand-tuned to retain both gun rights and gun control videos from a random sample of videos.

B. Minimum Wage. Our procedure for designing these studies largely followed that of the gun-rights study, with some modifications. For feasibility, these experiments used videos up to 12 minutes long. As before, we coarsely screened for topicality by applying a regular-expression filter to their titles. For videos passing this initial topicality screening, we extracted textual transcripts to classify for ideological valence. MTurk workers manually coded all videos. For each video classification task, we assigned three workers and labeled the videos following the 2/3 majority opinion. We saw a very high inter-coder agreement rate (on average 80% to 85% across multiple rounds of classification). Then, we filtered out videos that did not have a clear ideological orientation: only videos that supported or opposed raising the minimum wage appear in the final recommendation trees. Finally, authors conducted an additional round of classification on approximately 500 videos to validate the MTurk results. These steps resulted in a smaller sub-graph of around 1,090 unique videos with a binary label and are less than 12 minutes in length.

3. Experimental Implementation and Preregistration Details

We preregistered all four of our studies ahead of fielding each respective one. We preregistered Study 1 on Tuesday, June 8, 2021 just before beginning to field Wave 1 of the survey. Wave 1 recruited 3,902 participants (with the last coming in on Tuesday, June 15) which was a smaller number of participants than initially intended. In order to increase participation, survey compensation was raised to $2 from $1.50 for later waves of participants and we lifted the quota on political views. We posted a revised pre-analysis plan on Thursday, June 17, 2021, immediately before inviting 2,862 respondents back for Wave 2. This was approximately two days later than initially intended. We posted Wave 3 on Friday, June 25, 2021 and closed on Friday, July 2, 2021.

Study 2 (MTurk) and Study 3 (YouGov) were fielded starting May 2022. Wave 1 was fielded starting May 16 on MTurk and May 18 on YouGov. The PAP was posted at OSF just before data collection began for Wave 2 (before randomization and outcome data collection) on May 24. Wave 2 began fielding on May 25 for MTurk and May 26 for YouGov. Participants were paid $1.50 for completing the initial wave and $5 for completing Wave 2 (those in the pure control group received only $1 for Wave 2).

Finally, we posted a pre-analysis plan for the single-wave Study 4 and the "First Impressions" experiment on Wednesday, May 22, 2024, and began data collection for the "First Impressions" experiment on Thursday, May 23, 2024. Due to a typographic issue in the Study 4 survey, as well as the realization that participants were taking longer to complete the survey than anticipated, we posted an amended pre-analysis plan on May 23 and began data collection for Study 4 on Tuesday, May 28, 2024. The study closed on Friday, May 29, 2024.

Despite our attempts to recruit equal proportions of liberals and conservatives, our sample is somewhat skewed in terms of ideological self-placement (59% liberals and 30% conservatives including leaners) and partisan identification (63% Democrats and 27% Republicans including leaners). Well-known biases in terms of age distribution on MTurk are also present (20% under 30, 50% 30–44, and only 5% age 65 or older), though this arguably accords with the target population of frequent streaming video platform users.[§] Since partisanship is not completely predictive of gun attitudes, we still obtain substantial variation in our pre-treatment gun policy measure, though the distribution is still somewhat right-skewed (mean 0.41, median 0.35 on a 0–1 scale).

The demographics of our four survey samples were relatively similar and are shown in the four panels of Figure S1. In addition, Tables S1, S2, and S3 show these descriptive features of Studies 1–3 in tabular format.

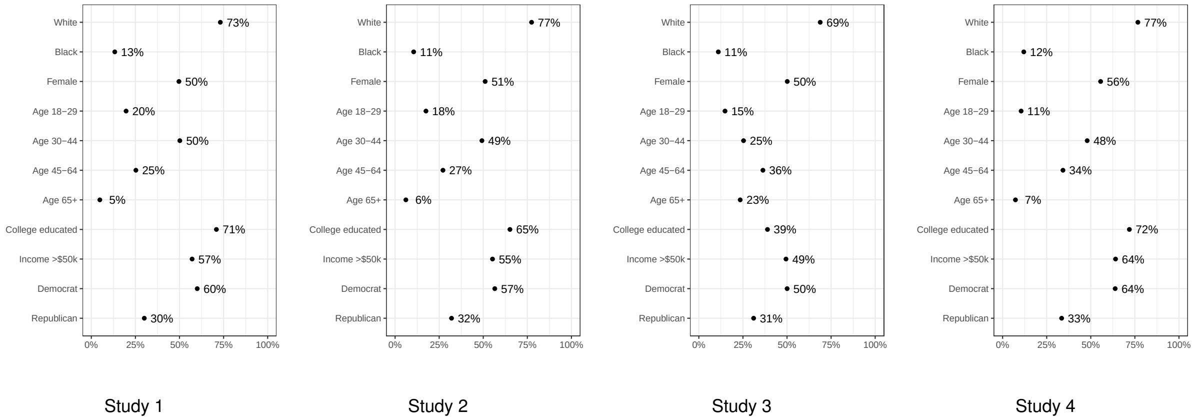

Fig. S1. Respondent Demographics

[§] See https://www.pewresearch.org/internet/fact-sheet/social-media/ for self-reported YouTube use by age category. See Figure S1 and Tables S1, S2, and S3.

Statistic	Mean	St. Dev.	Median	Min	Max	N
Female	0.50	0.50	0.00	0.00	1.00	3,904
White	0.73	0.44	1.00	0.00	1.00	3,903
Black	0.13	0.34	0.00	0.00	1.00	3,903
Age	39.94	12.25	37.00	18.00	84.00	3,903
College educated	0.71	0.45	1.00	0.00	1.00	3,904
Income >50k	0.57	0.49	1.00	0.00	1.00	3,902

Table S1. Study 1 Survey Respondent Demographics (Wave 1)

Statistic	Mean	St. Dev.	Median	Min	Max	N
Female	0.51	0.50	1.00	0.00	1.00	3,095
White	0.77	0.42	1.00	0.00	1.00	3,094
Black	0.11	0.31	0.00	0.00	1.00	3,094
Age	41.10	12.58	39.00	19.00	98.00	3,095
College educated	0.65	0.48	1.00	0.00	1.00	3,094
Income >50k	0.55	0.50	1.00	0.00	1.00	3,095

Table S2. Study 2 Survey Respondent Demographics (Wave 1)

Statistic	Mean	St. Dev.	Median	Min	Max	N
Female	0.50	0.50	1	0	1	4,591
White	0.69	0.46	1	0	1	4,591
Black	0.11	0.31	0	0	1	4,591
Age	50.29	16.94	52	19	94	4,591
College educated	0.39	0.49	0	0	1	4,591
Income >50k	0.49	0.50	0	0	1	4,591

Table S3. Study 3 Survey Respondent Demographics (Wave 1)

We also explore the amount of time respondents spent on the video interface. Looking at time in the video interface, we find that participants spent substantial time engaging with our stimuli. Study 1 had a median watch time of 12 minutes and a mean of 15 minutes; study 2 had a median of 24 minutes and a mean of 24 minutes; study 3 had a median of 27 minutes and a mean of 29 minutes; and study 4 had a median of 21 minutes and a mean of 19 minutes. Figure S2 plots the full distributions of time taken on the video interface.

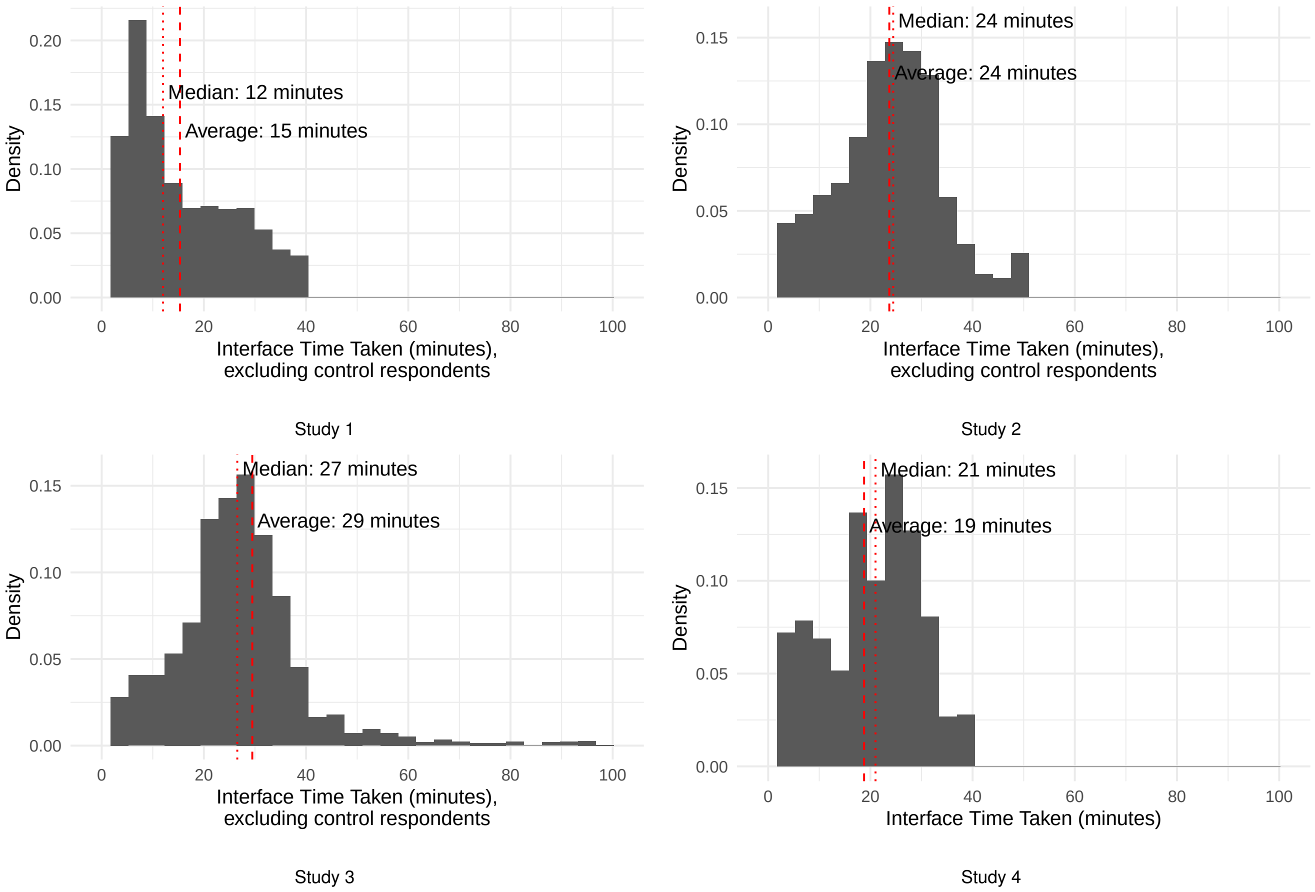

Fig. S2. Time taken by respondents on video platform.

4. Similarity to Browser-Based Recommendations

To our knowledge, there is no formal documentation explaining the relationship between the recommendations obtained from the YouTube API and those that are shown to actual users in the web or app interface. To investigate this, we conducted a validation exercise comparing API recommendations to those presented on the YouTube web interface in actual browser sessions to an anonymous user, both starting from the same video. We describe the conclusions in detail below; to summarize, we found that aside from some instances in which the web interface deviated to off-topic recommendations that would have been eliminated by our trimming procedure, the two sets of recommendations are largely the same.

To demonstrate the validity of our recommendation trees, which were created with the YouTube API, we create browser-based recommendation trees. To do so, we load a single seed URL in an automated anonymous browser. We record the 20 recommended videos for that seed video, then load each recommended video in separate anonymous browsers (to avoid dependencies created by the ordered history of viewing previous videos). We compare these recommendations to a tree concurrently created via the API, which is the method by which we created the trees used in our experiments (the trees used in the experiment were further filtered on topic according to the text of the video).

Both the API- and browser-based recommendations start with a single seed video: $15 minimum wage would cut 1.4 million jobs by 2025: CBO. We build the API-based recommendation network by taking three steps, recording 50 recommendations for each video in each step. In other words, at the first step, we collect the 50 videos recommended from our single seed video. In the second step, we record the 50 recommended videos for each of those 50 videos, and so on. (Deeper experimental recommendation trees can be constructed using this three-step API data collection, as the YouTube recommendation network is quite dense and contains numerous recommendations between videos collected in different steps.)

However, when loading YouTube in a browser, 20 recommended videos are visible in the browser. It is possible to get additional recommendations by scrolling down, but doing so massively slows down data collection and increases the chances of connection errors. As a result, in our browser-based tree, we collect 20 recommended videos at each node in the tree. Additionally, instead of taking 3 steps, we take 5. We compare these two trees and find that they are largely similar (Figure S3).

To get a better sense of why some recommendations are in the natural tree and not in the API tree, we manually inspect 10 randomly selected recommendations. At a high level, we found that differences were primarily driven by a larger share of off-topic recommendations in the browser, compared to the API. Figure 4 shows ten randomly selected watch sequences in the browser-based tree. The column farthest on the right shows the origin video (the same for all branches), the second shows the first recommendation in that branch, and so on for five steps. The cell values are the video ID of the YouTube video, and $***$ indicates that that particular video was *not* found in the API tree.

This table highlights several features of this exercise. First, because each video was inspected in a history-less browser, if browser recommendations branched off-topic, we found that the browser never returned to on-topic videos and so no subsequent recommendations are also found in the API tree. Therefore, the most nodes that contribute most to browser-based and API tree differences are those in which the browser recommendations deviate off topic. To verify that that is in fact what drives differences (as opposed to the browser-based tree recommending on-topic videos that are simply different than those found in the API tree), we closely examined these videos. They are as follows.

Two branches go off topic in the first step, video `Mqn4lYunTX4`. This is a 25-minute video titled "Bone in vs Boneless Steaks (How to be a Steak Expert) The Bearded Butchers."

One additional branch goes off topic in the second step: `wx_72QJTDUs`: "Chris Stapleton: The 60 Minutes Interview."

Three additional branches go off-topic in the third step: `0Q9zng2S810` ("Why North Korea is the Hardest Country to Escape"), `da1vvigy5tQ` ("Reversing Type 2 diabetes starts with ignoring the guidelines | Sarah Hallberg | TEDxPurdueU"), and `TLcw2xsQh68` ("Here's How Larger 34-Inch Off-Road Tires Affect My Ford F-150 Hybrid's MPG and 0-60 MPH Speed!").

By the fourth step, all but one of our sampled watch sequences have gone off topic. The recommendations that diverge at step four are `wANiIPO9TiQ` ("Target packaging Tiktok compilation • part 1"), `fKME33GDFZI` ("What to expect next... out of underwriting & Closing Disclosures (CD)"), and `i2trJEIFIvY` ("Why does maths give humans the edge over machines? - with Junaid Mubeen").

In the final step, the last on-topic branch goes off topic with `e0LBJfCTCo8`: "CNBC's Courtney Reagan reports on the groundbreaking life of the late Queen Elizabeth."

Fifth Step	Fourth Step	Third Step	Second Step	First Step	Seed Video
ISaZduGmhEU***	TtzsU4WAJ-k***	ph0yUhz-73U***	yomerhQkpSc***	Mqn4lYunTX4***	2voNlYS-8C0
e0LBJfCTCo8***	aqpr0uRsmcs	nLDtZN1dPHk	AtjaRuGkbgQ	RPwqBsc4Ffo	2voNlYS-8C0
7UAoT21eqXI***	RWQKa4qTbkE***	zRWvWe08HTA***	WZRvRbzTU_c***	Mqn4lYunTX4***	2voNlYS-8C0
e7Tao1t0i7E***	wANiIPO9TiQ***	oCmLhc1HNSI	aTVfbSeeS74	3-KMXng5Cp0	2voNlYS-8C0
wngB9_6Vqbc***	C0kWjEYMAfc***	0Q9zng2S810***	lwYOJLGw-Mw	zKyWRRJQbkM	2voNlYS-8C0
auw4Z6Ff0T4***	fKME33GDFZI***	C7PfqazmSuQ	FPLcO0kFhP0	UdnkStBTG2k	2voNlYS-8C0
d5wfMNNr3ak***	4lzs5wpLkeA***	da1vvigy5tQ***	S1E8SQde5rk	Hatav_Rdnno	2voNlYS-8C0
63slKb4iGO8***	VUlRz2ih1uc***	TLcw2xsQh68***	-e55Vued028	Hatav_Rdnno	2voNlYS-8C0
wx_72QJTDUs***	GayEgDB1EZY***	kAE3F-35OP0***	wx_72QJTDUs***	wqKfL3z5yM4	2voNlYS-8C0
7dzoGb-jcW4***	i2trJEIFIvY***	ZuXzvjBYW8A	QaN6ibm5r-I	8H4yp8Fbi-Y	2voNlYS-8C0

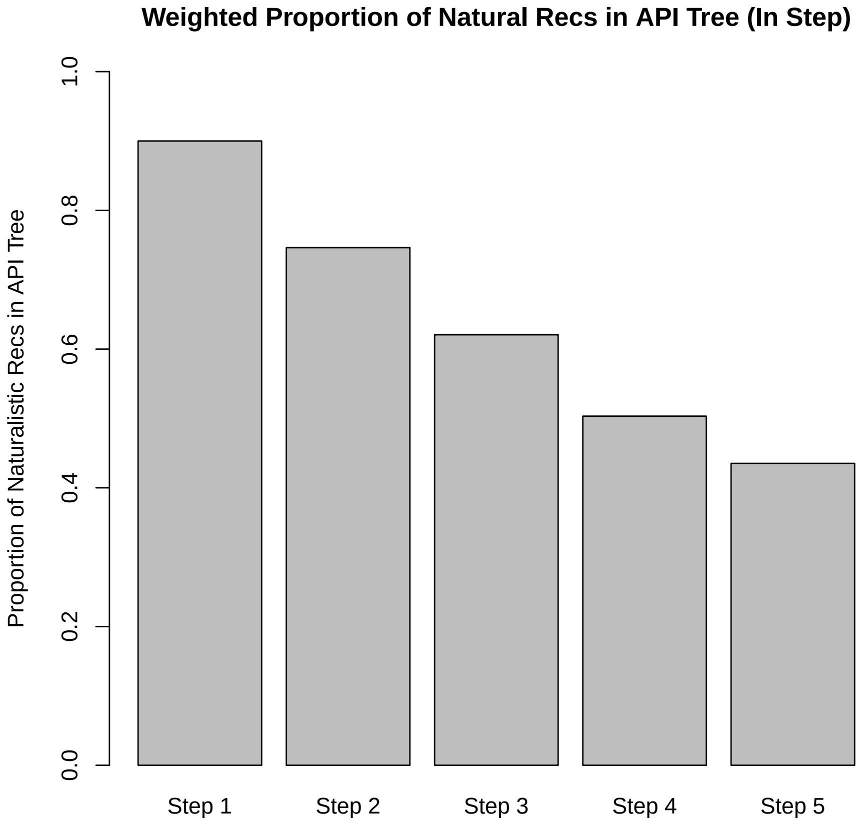

Fig. S3. A comparison between the trees created for our experiments (via the API), and naturalistic recommendations scraped directly from YouTube. The x-axis shows each "step" of the natural recommendation tree obtained from YouTube. We observe that, especially in earlier steps, the majority of the video recommendations that appear in the naturalistic trees also appear in our experiments, which provides reassuring evidence that the experimental trees capture real recommendation patterns from YouTube. However, as one traverses deeper into the browser tree, the videos have a tendency to deviate from the topic of interest, which contributes to the lower proportion of present recommendations in later steps.

5. MTurk HIT Recruitment Language (Study 1, Wave 1)

Title: Participate in a Streaming Video Study (5–10 minutes)

Description: We're interested in learning how people like you respond to videos shown on an interactive interface. In this initial survey, we would like to learn more about your video habits and background. We may invite you to use a video platform in a future study.

What is this study about? We designed an interactive streaming video interface to present videos about a topic and adapt to your preferences. We are interested in learning how people like you respond to videos and what you remember from the experience.

What is the problem being solved by this study? How to discover and rank content (videos in this case) from the vast quantities available online is a difficult question. We would like to explore how best to present information that is both high quality and relevant to users' interests.

How might research in this area change society? What users demand and what is good collectively for society may not always align. We are broadly interested in understanding the consequences of different ranking approaches on key democratic outcomes. We hope our results will inform decisions by social platforms that increasingly structure our informational choices.

What does it involve? This initial task involves answering a few questions about yourself, including your video watching habits and preferences. Sound and video are required! We may follow up with you and invite you to use our streaming video platform and to answer another set of questions, for additional compensation.

To participate, please open the following survey (8-10 minutes) in a new tab or window.

As suggested, this initial survey will determine eligibility for a future study (with additional compensation) that will involve an interactive, streaming video interface.

6. MTurk HIT Recruitment Language (First Impressions Experiment)

HIT Title: Guess Content of Videos from Their Thumbnails (<5 minutes) The goal of this activity is to *guess the content of a video based only on the thumbnail.* We are interested in how accurately you are able to predict what the videos are about, even without watching the video. You will be presented with approximately 20 thumbnails, and you will have to decide between multiple options when guessing its content.

To incentivize you to choose accurately, you will be paid a **5 cent bonus** for each video thumbnail that you get correct, on top of your base pay.

Keywords: YouTube, video, thumbnail, guessing, content

7. MTurk HIT Recruitment Language (Study 4; "Rabbit Hole" Experiment)

HIT Title: Participate in a Video Streaming Study (15-45 minutes) We're interested in learning how people like you respond to videos shown on an interactive interface. We designed an interactive streaming video interface to present videos about a topic. You will also have to answer a few questions about yourself, including your video watching habits and preferences. Sound and video are required!

Keywords: YouTube, video, watching, audio, sound, topics, survey

8. Survey Question Wording

A. Policy Attitudes.

A.1. Study 1: Gun Control. In study 1, our primary outcome of interest was an additive index ranging from 0 to 1 formed from a five-question battery of gun policy attitudinal questions. These questions were adapted from common question wordings placed on national surveys run by Pew, Gallup, the *Washington Post*, and other policy attitude surveys. We show these individual questions below:

1. What do you think is more important — to protect the right of Americans to own guns, or to regulate gun ownership?
    - Protect the right to own guns
    - Regulate gun ownership
2. Do you support or oppose a nationwide ban on the sale of assault weapons?
    - Strongly support
    - Somewhat support
    - Neither support nor oppose
    - Somewhat oppose
    - Strongly oppose
3. Do you support or oppose a nationwide ban on the possession of handguns?
    - Strongly support
    - Somewhat support
    - Neither support nor oppose
    - Somewhat oppose
    - Strongly oppose
4. Suppose more Americans were allowed to carry concealed weapons if they passed a criminal background check and training course. If more Americans carried concealed weapons, would the United States be safer or less safe?
    - Much safer
    - Somewhat safer
    - No difference
    - Somewhat less safe
    - Much less safe
5. Do you support or oppose stricter gun control laws in the United States?
    - Strongly support
    - Somewhat support
    - Neither support nor oppose
    - Somewhat oppose
    - Strongly oppose

We rescaled each item to a unit scale, with 0 representing the most liberal of the response options and 1 representing the most conservative of the response options (i.e. reverse coding questions 1 and 4) for each question. Using principal components analysis, we found a Cronbach's α of 0.92 for the five-item scale, suggesting that all five items load on the same factor. In the appendix of our resulting manuscript we will report the results of an exploratory factor analysis with varimax rotation of these five attitudinal questions to verify that they load on the same underlying dimension. We then averaged the rescaled outcomes from all five questions to form the additive index such that the index has a range from 0 to 1.

A.2. Studies 2–4: Minimum Wage. In studies 2 and 3, our primary outcomes of interest were an additive index ranging from 0 to 1 formed from a five-question battery of attitudinal questions about minimum wage policy. These questions were, similar to our questions from study 1, adapted from common question wordings placed on national surveys. Following an anchoring baseline page that stated "As you may know, the current federal minimum wage is $7.25 an hour," we asked the following individual questions:

1. What do you think the federal minimum wage should be? Please enter an amount between $0.00 and $25.00 in the text box below.
    - ______________
2. Some people believe that raising the minimum wage would overly restrict the freedom of businesses to set their own employment policies. Imagine those people are all the way at one end of a scale, at 1. Other people might believe that raising the minimum wage protects workers from businesses exploiting workers. Imagine those people are at the other end of the scale, at 10. Of course, some people fall in between and believe that raising the minimum wage might or might not protect workers from businesses. Where would you place yourself on this scale?
    (a) Would restrict businesses' freedom
    (b)
    (c)
    (d)
    (e)
    (f)
    (g)
    (h)
    (i)
    (j)
    (k) Would protect workers from exploitation
3. Some people believe that raising the minimum wage would help low-income workers get by. Imagine those people are all the way at one end of a scale, at 1. Other people might believe that raising the minimum wage would hurt low-income workers. Imagine those people are at the other end of the scale, at 10. Of course, some people fall in between and believe that raising the minimum wage might or might not hurt low-income workers. Where would you place yourself on this scale?
    (a) Would help low-income workers
    (b)
    (c)
    (d)
    (e)
    (f)
    (g)
    (h)
    (i)
    (j)
    (k) Would hurt low-income workers
4. How high do you think the federal minimum wage should be?
    - Much higher than the current level
    - Somewhat higher than the current level
    - About the current level
    - Somewhat lower than the current level
    - Much lower than the current level
5. Do you support or oppose raising the federal minimum wage?

- Strongly support raising the minimum wage
- Somewhat support raising the minimum wage
- Neither support nor oppose raising the minimum wage
- Somewhat oppose raising the minimum wage
- Strongly oppose raising the minimum wage

6. The Raise the Wage Act is a proposal to raise the minimum wage so that it would be increased to $15 per hour by 2025. Do you support or oppose the Raise the Wage Act?
    - Strongly support
    - Somewhat support
    - Neither support nor oppose
    - Somewhat oppose
    - Strongly oppose
7. The Raise the Wage Act is a proposal to gradually raise the minimum wage. The minimum wage would first be increased to $9.50 an hour in 2022. Then, it would be increased by $1.50 an hour or less every year through 2025. Do you support or oppose the Raise the Wage Act?
    - Strongly support
    - Somewhat support
    - Neither support nor oppose
    - Somewhat oppose
    - Strongly oppose
8. How strongly do you support or oppose a $15 minimum wage?
    - Strongly support
    - Somewhat support
    - Neither support nor oppose
    - Somewhat oppose
    - Strongly oppose

Similar to Study 1, in Studies 2 and 3 we rescaled each item to a unit scale, with 0 representing the most liberal of the response options and 1 representing the most conservative of the response options for each question. For question 1, we rescaled respondents' numeric entries such that $25/hour was the most liberal response option and $0 was the most conservative option.¶ Using principal components analysis, we found a Cronbach's α for the eight-item scale of 0.94 in Study 2 and 0.94 for Study 3, suggesting that all eight items load on the same factor. We then averaged the rescaled outcomes from all eight questions to form the additive index such that the index has a range from 0 to 1.

A.3. Study 4: Minimum Wage. Study 4 uses a very similar version of the Minimum Wage Policy Index as in Studies 2 and 3, with a modification of 2 questions (#6 and #7) related to the Raise the Wage Act (which was, at the time of conducting Study 4, outdated). The wording for the two questions were revised to the following, based on the latest-proposed version of the Raise the Wage Act:

1. The Raise the Wage Act is a proposal to raise the minimum wage so that it would be increased to $17 per hour by 2028. Do you support or oppose the Raise the Wage Act?
    - Strongly support
    - Somewhat support
    - Neither support nor oppose
    - Somewhat oppose
    - Strongly oppose
2. The Raise the Wage Act is a proposal to gradually raise the minimum wage. The minimum wage would first be increased to $9.50 an hour by 2024. Then, it would be increased by $1.50 an hour or less every year through 2028. Do you support or oppose the Raise the Wage Act?

¶ We omit any answers that respondents gave that were over $25/hour.

- Strongly support
- Somewhat support
- Neither support nor oppose
- Somewhat oppose
- Strongly oppose

A.4. Media Trust/Hostility. In order to measure effects on media trust/hostility, in all four studies we asked two questions about beliefs in fabricating news stories, both by major news organizations and YouTube channels, shown below.

1. Based on what you know, how often do you believe the nation's major news organizations fabricate news stories?
    - All the time
    - Most of the time
    - About half the time
    - Once in a while
    - Never
2. Based on what you know, how often do you believe YouTube channels fabricate news stories?
    - All the time
    - Most of the time
    - About half the time
    - Once in a while
    - Never

As an additional measure of media trust, we used a grid question which asked respondents to rate how much, if at all, they trust the information they get from several media sources. Specifically, this grid asked about trust in information from major news organizations, local news outlets, social media, and YouTube. Response options were: A lot, Some, Not too much, and Not at all. We examined effects on both trust in major news organizations and in YouTube.

A.5. Affective Polarization. Our fourth family of outcomes for all three studies measured respondents' affective polarization using several standard questions for this concept. First, we used a pair of questions (shown below) that asked respondents how smart people are who support the party the respondent prefers vs. the other party (1–5 where 5 indicates "extremely" smart for both). This outcome measure was calculated as the difference in perceptions between the ingroup question and the outgroup question. While the results were collected for respondents who did not indicate a preference for or lean towards a political party (i.e. "pure independents"), we did not use these responses.

1. In general, how smart are people who support Democrats?
    - Extremely
    - Very
    - Somewhat
    - A little
    - Not at all
2. In general, how smart are people who support Republicans?
    - Extremely
    - Very
    - Somewhat
    - A little
    - Not at all

Second, we looked at the difference between the feeling thermometer scores respondents assigned to the outparty vs. the inparty. Finally, we measured the difference between responses on two questions about comfort with having members of the inparty vs. outparty as close personal friends, shown below (same conditions on pure independents apply for these measures):

1. How comfortable are you having close personal friends who are Democrats?

- Not at all comfortable
- Not too comfortable
- Somewhat comfortable
- Extremely comfortable

2. How comfortable are you having close personal friends who are Republicans?

- Not at all comfortable
- Not too comfortable
- Somewhat comfortable
- Extremely comfortable

9. Details of Study Design and Analysis

The policy-attitude questions comprising our primary outcome index were quite reliable: for Study 1, $\alpha = 0.87$; study 2, $\alpha = 0.94$; study 3, $\alpha = 0.94$; and study 4, $\alpha = 0.94$. We also pre-registered an exploratory factor analysis with varimax rotation for these questions. The proportions of variance explained by a single dimension are 0.68, 0.72, 0.73, and 0.73. respectively. Our media-trust questions were taken from standard batteries used in research on political communication (e.g. 1), while our measures of affective polarization were similarly taken from validated measures of out-party animosity (e.g. 2).

Following our pre-registration, we assessed the effects of the video recommendation algorithm by comparing the post-treatment attitudes of respondents in different experimental conditions, based on the same liberal-ideologue, moderate, and conservative-ideologue subgroups used in treatment assignment. We analyzed post-treatment attitudes using regressions that controlled for a set of attitudes and demographic characteristics that were measured pre-treatment per our pre-analysis plan. Our main analyses examined the effect of the slanted recommendation algorithm (vs. the balanced algorithm) on respondents' video choices; their platform interactions; and their survey-reported policy attitudes, media trust, and affective polarization. Specifically, in the policy-attitude, media-trust, and affective-polarization analyses, we control for pre-treatment versions of all outcomes in the hypothesis family, defined below. In the platform-interaction analyses, we control for age, gender, political interest, YouTube usage frequency, number of favorite YouTube channels, whether popular YouTube channels are followed, text/video media consumption preference, a self-reported gun enthusiasm index, and perceived importance of the gun policy issue. We pre-registered the use of the Lin (3) estimator (using demeaned controls, all interacted with treatment) but found this to produce an infeasible number of parameters. As a result, we instead use controls in an additive (non-interacted) regression with robust standard errors. These results are substantively similar to the unadjusted results. Study 1 and the MTurk sample for Study 2 contained an additional "pure control" condition that involved watching no videos. Per our pre-registration, we committed to only using this control condition if there was a newsworthy event related to the policy issue under study, which did not occur during either study. We conducted stratified randomization to these experimental conditions based on respondents' pre-treatment political attitudes on the policy subject. Respondents in the most liberal tercile ("liberal ideologues") were only shown a liberal seed video, meaning that the only randomization for these subjects was between the balanced and slanted recommendation algorithm. This avoided forcibly exposing liberal participants to conservative viewpoints that they did not voluntarily consume, improving the realism of the study. Similarly, "conservative ideologues" initially in the most conservative tercile were only exposed to conservative seed videos. "Moderate" respondents, defined as the middle tercile of pre-treatment attitudes, were randomly presented with either liberal or conservative seed videos.

We examine three layers of hypotheses: (1) whether the experiment had any effect on a family of outcomes, broadly construed; (2) which subgroup and treatment contrast generates the effect; and (3) the specific outcome on which the effect manifests. The correction proceeds as follows. Within hypothesis families that survive the first-stage assessment of overall significance, we proceed to disaggregated examination of individual hypotheses. The initial "layer-1" family-level filtering is conducted using Simes' method (4) to combine layer-2 p-values (defined below) across the six treatment contrasts. This tests the intersection null that no version of the treatment had any effect on any outcome in the family. Because four hypothesis families are tested, an additional Benjamini-Hochberg (BH) correction (5) is applied to the family's Simes p-value before interpreting the layer-1 results. We say that a family "survives" if its BH-corrected Simes p-value is less than 0.05. Within each hypothesis family and treatment contrast, layer-2 p-values are obtained by an F-test from a multiple-outcome regression, testing the null that the contrasted treatment groups are identical on all outcomes in the family. (If an F-test for joint significance cannot be computed for the multiple-outcome regression due to numerical issues in the variance-covariance matrix, we will fall back on an alternative, more conservative procedure in which we conduct separate regressions for each outcome and combine them with the Simes method.) We only seek to interpret a family's layer-2 p-values (which correspond to specific treatment contrasts) if the family survives layer-1 filtering (indicating that some effect exists for some treatment contrast). To interpret layer-2 p-values, we first apply a BH correction to the F-test results, then multiply by an additional inflation factor (one over the proportion of surviving families) to account for selection at layer 1. Finally, for treatment contrasts that survive layer-2 filtering, we examine which specific outcomes in the family are affected. These layer-3 p-values are obtained by disaggregating the previous analysis into single-outcome regressions. As before, a BH correction is applied to account for the fact that multiple outcomes are evaluated; in addition, inflation factors for layer-1 and layer-2 selection are also applied.

10. Main Results (Study 1 - 3)

Each of the figures presented in this section contains four panels, depicting results for four families of dependent outcomes: (1) participants' survey-reported policy attitudes; (2) their platform interactions; (3) survey-reported media trust, and (4) survey-reported affective polarization. The top two panels are included in the corresponding version of the figure in the main text.

Figure S4 corresponds to Figure 4 in the main text, and shows the effect of different recommendation algorithms among conservative ideologues. We find that conservative ideologues do not change their policy attitudes in Studies 1 and 2, but that they shift towards slightly more conservative views in Study 3 (the YouGov sample on the Minimum Wage question). Additionally, we find that a slanted (3/1) recommendation system led conservative ideologues to select more conservative videos, but there were no discernible effects on policy attitudes (media trust and affective polarization).

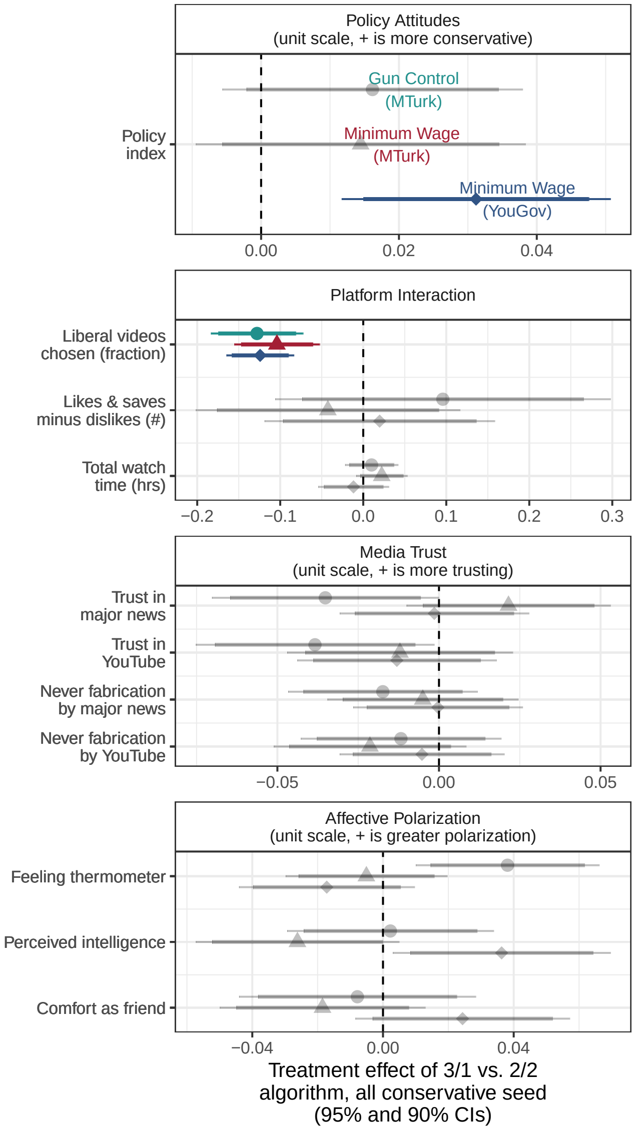

Fig. S4. Effects of recommendation algorithm among conservative ideologues. Displays the effects of more algorithmic recommendation slant (vs. balance) on behaviors and attitudes among conservative ideologues (those in the third tercile of pre-treatment policy attitudes). Gray points and error bars represent estimated effects that are not statistically significant after implementing multiple testing corrections, while points and error bars in color represent those effects that are still statistically significant after multiple testing corrections. See complete results in SI 10.

Figures S5, S6 S7, and S8 show our primary results among moderate participants (expanding on Figures 5 – 8 in the main text). As in the corresponding Figures 5 – 6, Figures S5 and S6 show algorithmic effects among moderate participants, with Figure S5 showing the effect of a slanted recommendation system among moderates assigned to a liberal seed and Figure S6 showing the effect of a slanted recommendation among moderates assigned to a conservative seed. Overall, we find that moderates choose to watch more videos of the seed that they were assigned to (that is, those who are assigned to a liberal seed watch more liberal videos, and vice versa for those assigned to a conservative seed). In Study 3, we also observe that the

slanted algorithm (3/1) causes moderate participants to spend 7.3 more minutes on the platform when they are assigned a liberal video, and in Study 1 the slanted algorithm causes them to spend 4.9 fewer minutes on the platform when they are assigned a conservative video. Nevertheless, we observe no changes in participants' media trust and affective polarization (the bottom two panels of the figures), and generally limited changes in policy attitudes. The only exception is that, in Study 3, moderates assigned to watch a conservative video reported slightly more conservative opinions post-treatment. Taken together, these results suggest that, despite changes in platform behaviors, the video treatments also had limited effects on moderate participants' political attitudes.

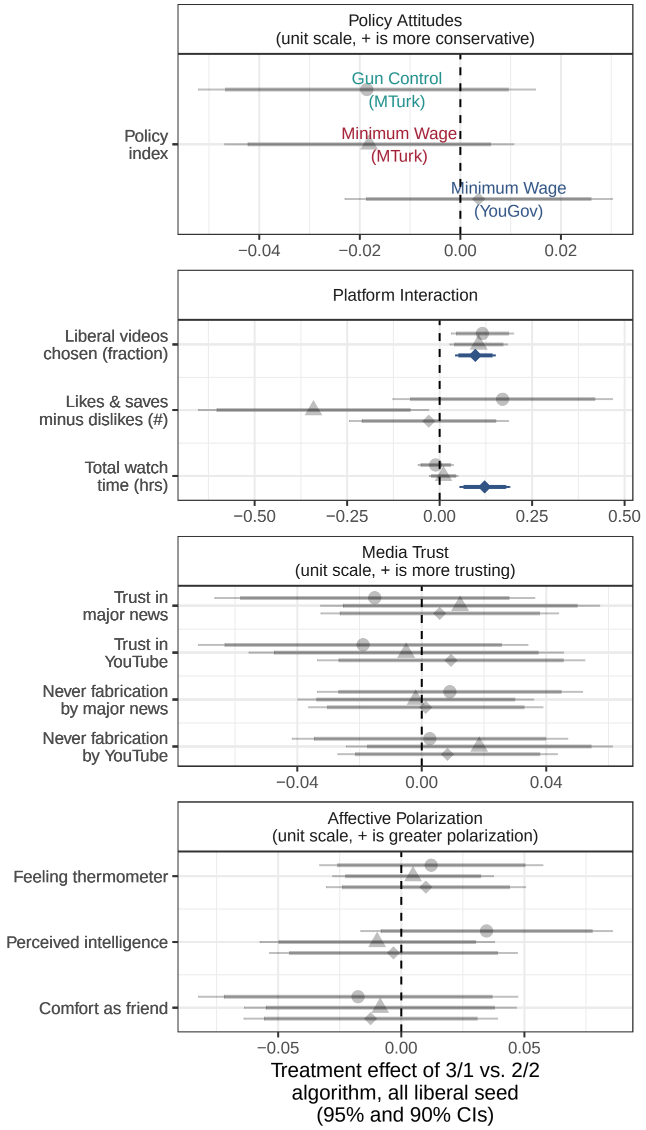

Fig. S5. Effects of recommendation algorithm among moderates assigned liberal seed video. The effects of more algorithmic recommendation extremity (vs. balance) on behaviors and attitudes among moderates (those in the middle tercile of pre-treatment policy attitudes) assigned to a liberal (i.e. pro-gun control or pro-minimum wage) seed video. Gray points and error bars represent estimated effects that are not statistically significant after implementing multiple testing corrections, while points and error bars in color represent those effects that are still statistically significant after multiple testing corrections.

As in the corresponding Figures 7 – 8, Figures S7 and S8 show forced-exposure effects among moderate participants who were assigned a conservative seed, with Figure S7 showing effects for those who were shown a slanted (3/1) mixture of recommended videos and Figure S8 showing effects for those assigned a balanced (2/2) mixed of recommendations. Moderates assigned to the conservative seed reported slightly more conservative policy attitudes post-treatment when assigned to the slanted (3/1) mixture of recommendations, but did not report discernibly different attitudes when assigned to the balanced (2/2) mixture of recommendations. Similarly, we also observe that participants assigned to the slanted (3/1) mixture tend to spend less time on the platform, and to make more conservative video choices. Again, however, we observe no significant differences in policy attitudes (bottom two panels of Figures S7 and S8).

Thus, while we observe some shifts in policy attitudes (particularly among moderates assigned to a slanted conservative

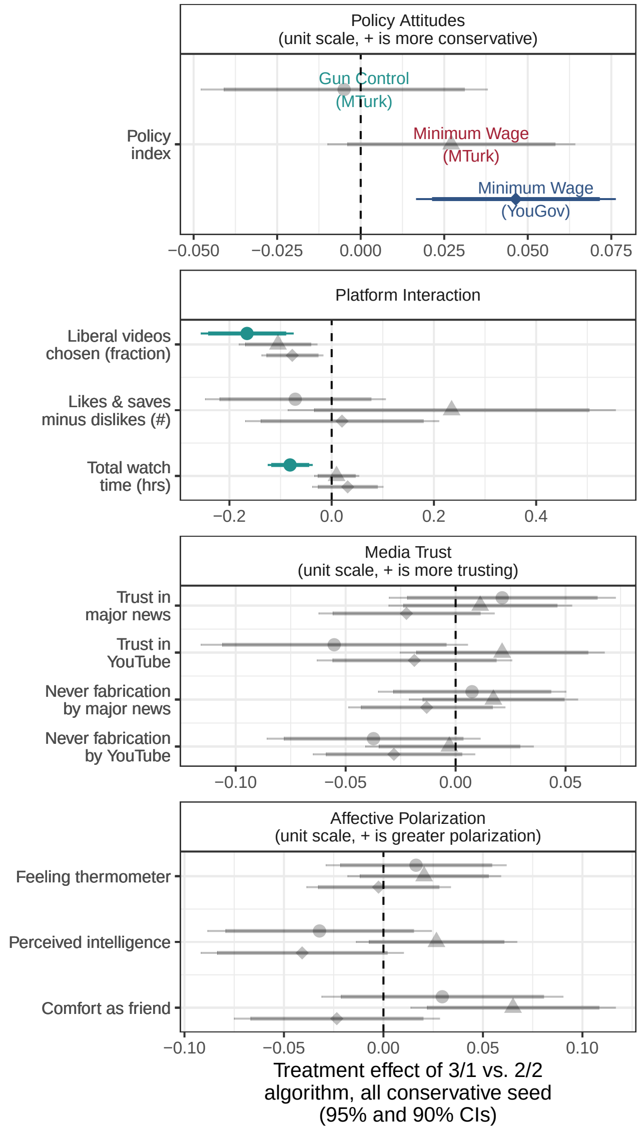

Fig. S6. Effects of recommendation algorithm among moderates assigned conservative seed video. The effects of more algorithmic recommendation extremity (vs. balance) on behaviors and attitudes among moderates (those in the middle tercile of pre-treatment policy attitudes) assigned to a conservative (i.e. anti-gun control or anti-minimum wage) seed video. Gray points and error bars represent estimated effects that are not statistically significant after implementing multiple testing corrections, while points and error bars in color represent those effects that are still statistically significant after multiple testing corrections.

seed), and while there are significant changes in participants' watching behaviors, none of these changes ultimately appear to impact participants media trust and affective polarization attitudes.

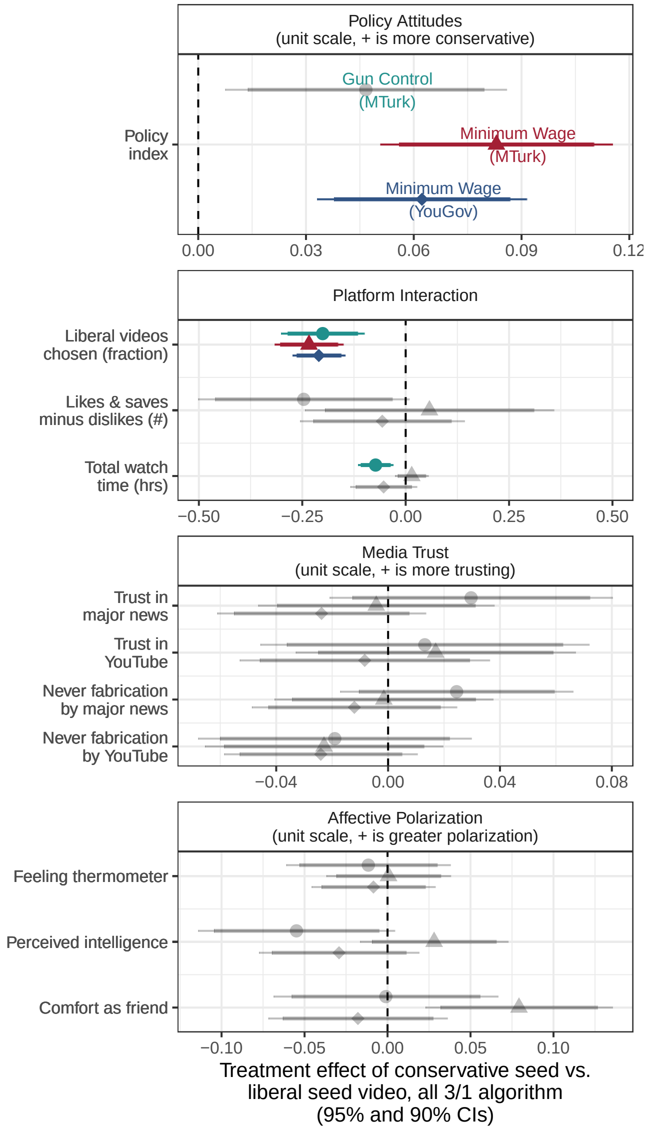

Fig. S7. Effects of seed video slant among moderates, 3/1 recommendation algorithm. The effects of a more conservative seed video on behaviors and attitudes among moderates (those in the middle tercile of pre-treatment attitudes) assigned to a 3/1 recommendation algorithm. Gray points and error bars represent estimated effects that are not statistically significant after implementing multiple testing corrections, while points and error bars in color represent those effects that are still statistically significant after multiple testing corrections.

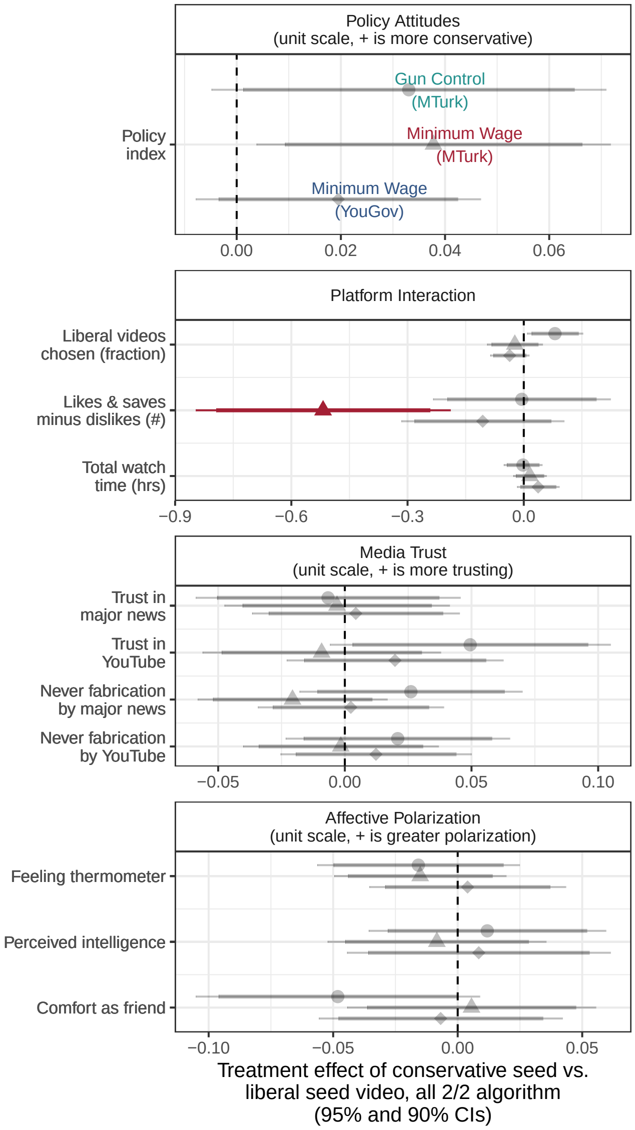

Fig. S8. Effects of seed video slant among moderates, 2/2 recommendation algorithm. The effects of a more conservative seed video on behaviors and attitudes among moderates (those in the middle tercile of pre-treatment attitudes) assigned to a 2/2 recommendation algorithm. Gray points and error bars represent estimated effects that are not statistically significant after implementing multiple testing corrections, while points and error bars in color represent those effects that are still statistically significant after multiple testing corrections.

11. Assessing Potential Changes in Issue Understanding or Interpretation in Studies 1–3

At the suggestion of a reviewer, we conducted additional analyses to assess whether our experimental manipulations might have shifted the way that participants understand or interpret a political debate—a possible precursor to persuasion that might manifest later—even though our overall results generally found precisely estimated null effects on their overall positions. In our primary analyses, this overall position was measured using an additive index of the questions described in SI A.1 and A.2, weighted equally and rescaled to the $[0, 1]$ interval. For these additional analyses, we extracted questions specifically relating to understandings and interpretations to analyze separately; these questions are summarized below for ease of reference. We caution that these analyses were not preregistered and should be regarded as exploratory.

- **Study 1 (Gun Rights):** "What do you think is more important — to protect the right of Americans to own guns, or to regulate gun ownership?"
- **Study 1 (Gun Rights):** "If more Americans carried concealed weapons, would the United States be safer or less safe?"
- **Studies 2–3 (Minimum Wage):** "Some people believe that raising the minimum wage would overly restrict the freedom of businesses to set their own employment policies. . . [vs.] Other people might believe that raising the minimum wage protects workers from businesses exploiting workers... Where would you place yourself on this scale?"
- **Studies 2–3 (Minimum Wage):** "Some people believe that raising the minimum wage would help low-income workers get by. . . [vs.] Other people might believe that raising the minimum wage would hurt low-income workers... Where would you place yourself on this scale?"

Methodologically, we modify the approach previously used to analyze the primary index outcome: for various subgroups of respondents, we regress the specific post-treatment attitude (instead of the overall policy index used in the main analysis) on a binary treatment-assignment indicator and the pre-treatment attitude. The regression specification is thus identical with the exception of a differing outcome. On minimum wage, we found significant effects of algorithmic slant in both Studies 2 and 3. When revisiting Study 2, we found that conservative respondents assigned to the slanted 3/1 algorithm (vs. the balanced 2/2) moved by +0.04 on a one-point scale, toward the belief that minimum wages "restrict the freedom of businesses". While initially significant, this result has $p = 0.057$ after multiple-testing corrections. In Study 3, again among conservative respondents only, we found that the slanted algorithm moved respondents by +0.05 toward the belief that minimum wages "hurt low-income workers" ($p = 0.010$ after multiple-testing correction). These were the only algorithmic effects that we observed. Both retained statistical significance after multiple testing corrections, though it is worth noting these algorithmic effects are roughly half the size of the traditional forced-exposure effects that we observe when randomizing initial seed video. Results for Study 1 were slightly weaker: while results initially suggested that conservative respondents moved +0.03 on the belief that more concealed weapons would make the U.S. safer, this result lost statistical significance after multiple-testing corrections. However, we do not find algorithmic effects on how moderates and liberals understand and interpret the policy issues that we study, which suggests the need for caution in drawing conclusions from these exploratory analyses and the need for future preregistered work. Our results are summarized in Figure S9.

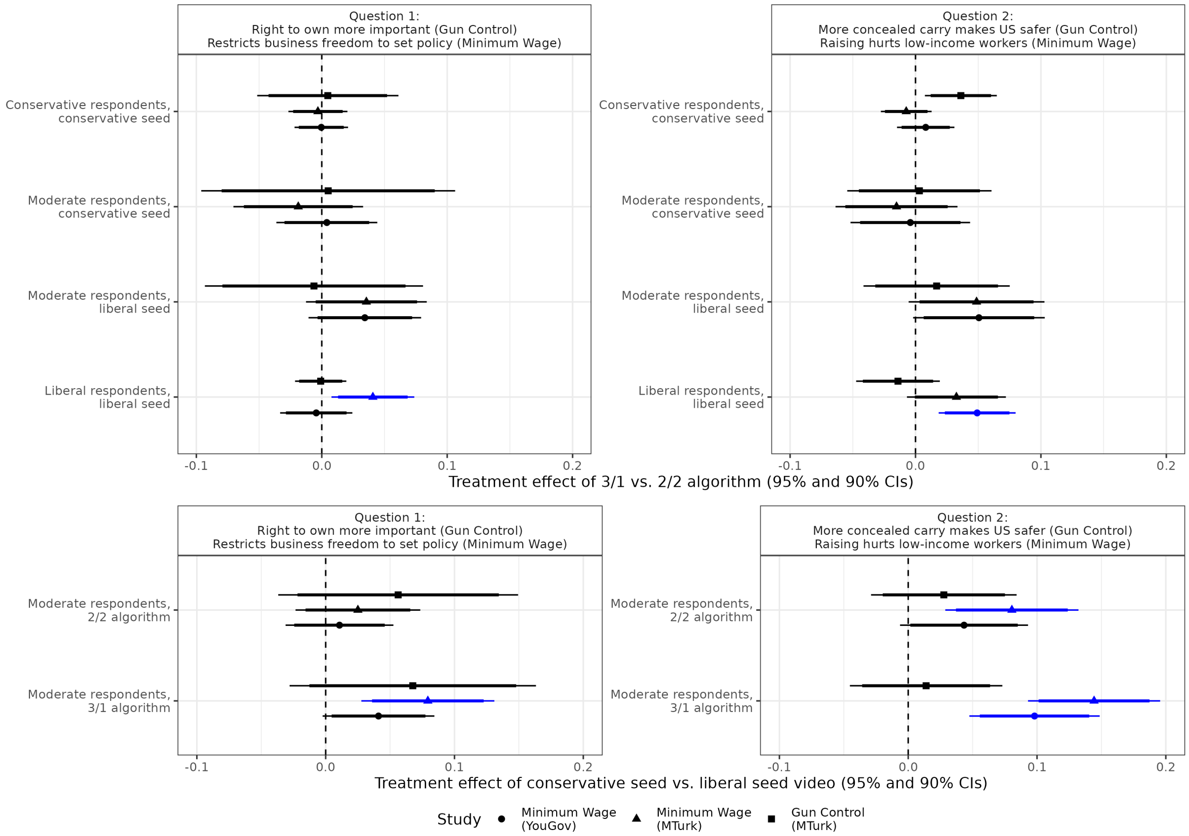

Fig. S9. Effects of recommendation algorithm and seed video slant on issue understandings and interpretations. The y-axis depicts various subgroups of participants, and the x-axis indicates treatment effects on participant responses (positive values represent more conservative attitudes). The top panels depict the effects of an algorithmic intervention that serves a slanted 3/1 mix of recommendations (versus a balanced 2/2 mix). The bottom panels represent effects of forced exposure interventions that deliver a conservative seed video (versus a liberal one). Left and right panels illustrate effects on different survey questions; note that “Question 1” and “Question 2” correspond to different questions in Study 1 and Studies 2–3. Grey points and error bars represent estimated effects that are not statistically significant after implementing multiple-testing corrections, while blue points and error bars represent those effects that are still significant after multiple-testing corrections.

12. Effect Heterogeneity in Studies 1–3

To assess potential effect heterogeneity, we tested for effect moderation along political interest, frequency of YouTube usage, college education, age, and gender. We binarized moderators by cutting them at the median (except for college education and gender). All tests of effect heterogeneity were preregistered apart from college education, which was added at the request of a reviewer.

Methodologically, we extended the approach of our primary attitudinal-change analyses: there, we regressed post-treatment policy attitudes on (1) a binary treatment-assignment indicator for those randomized into the balanced 3/1 algorithm, vs. the balanced 2/2 algorithm; and (2) a pre-treatment attitude measure. Our original analyses were repeated within four subgroups of comparable respondents: liberals, moderates assigned to start on a liberal video, moderates assigned to start on a conservative video, and conservatives.

In these heterogeneity analyses, we extended the original specification by adding a term for the moderator and a moderator-treatment interaction (we tested one moderator at a time). In total, we conducted 60 tests (3 studies x 4 subgroups x 5 moderators). Across these 60 tests, only three reached conventional levels of statistical significance, even prior to multiple-testing corrections. All were in the minimum-wage issue, and all were for male-female heterogeneity. Moreover, all three lose significance after a Benjamini-Hochberg multiple-testing correction. The factors that we initially regarded as more plausible (political interest, frequency of YouTube usage, college education, and age) did not significantly moderate the effect of slanted vs. balanced recommendation algorithms, even prior to multiple-testing corrections.

13. First-Impression Labeling

A. Motivation. The behavioral outcome of choice in Studies 1–3 implicitly assumes that, as participants are shown recommendations for left- versus right-leaning videos, the choice of which video to watch is informed by the perception of a video's political leaning from the recommendation page. Put another way, when participants are recommended a left- versus right-leaning video, decisions are made with at least some information about what they are choosing to watch.

The First-Impression Labeling Experiment tests this assumption. We provide participants with only the information on a standard recommendation page (the video's thumbnail image, title, channel, and number of views), and we ask participants to "guess" the political stance of the video. We then compare participants' guesses to the "ground truth" labels curated in the earlier experiments (Studies 1–3). This experiment serves as a manipulation check to ensure that participants are able to tell when they are being recommended more liberal or conservative videos—and that they know a video's general political stance when choosing among the recommendations.

B. Stimuli Overview. We included all unique videos that appeared in a recommendation tree in Studies 1–3, applying a minimal set of filters to improve data quality:

- We removed videos with missing recommendation page information (i.e., there was no valid video title, channel, or view count). This likely occurred because the relevant video had been removed from YouTube at some point after the original data was collected.
- We removed videos with invalid thumbnail images (i.e., a request to the image link resulted in a 404 server response), since these could not be shown to participants.
- (New) We removed 2 duplicated videos.
- (New) We removed 7 videos for which the ground truth label was missing.

The final two criteria, marked (New), were reasonable modifications made after we posted the Pre-Analysis Plan. Our final dataset consists of **72** gun control videos and **152** minimum wage videos.

C. Experimental Infrastructure. Participants are shown an interface that mimics a YouTube recommendation page, but that asks participants to evaluate each video using radio buttons below each thumbnail image (Figure S10). This design maintains ecological validity, as it asks participants to evaluate thumbnails in a context as similar as possible to that of the original experiment.

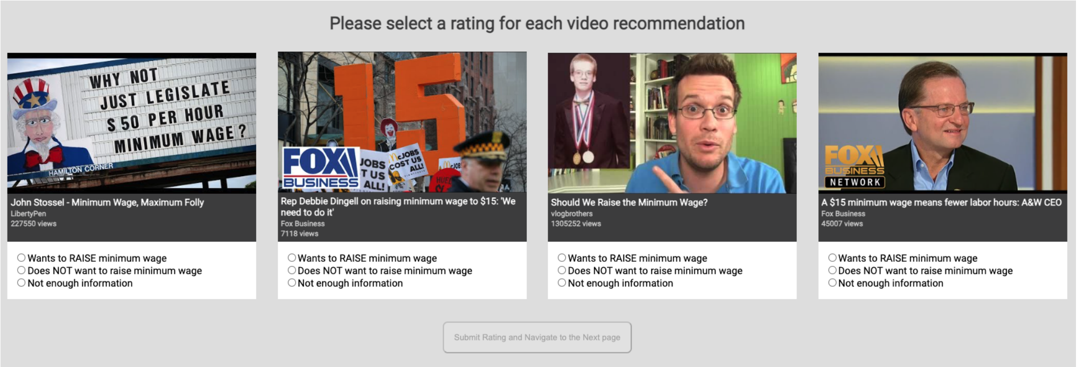

Fig. S10. First-Impression Labeling interface. The First-Impression Labeling interface mimics a YouTube recommendation page, and is based on the recommendation shown to participants in the original experiment. The primary difference is the radio buttons below each recommendation, in which participants are asked to evaluate the policy stance of each video. Participants must evaluate all four videos on the page before being allowed to proceed to the next page.

To make the evaluation of the thumbnails more concrete, participants are asked to make their assessments in terms of the policy position supported by the video. The language emphasizes the policies that the video is expected to support or oppose (e.g., "wants to raise the minimum wage") rather than asking participants to associate the policy with a partisan or ideological position.

The possible labels for the minimum wage thumbnails are the following:

- WANTS to raise the minimum wage
- Does NOT want to raise the minimum wage
- Not enough information

The possible labels for the gun control thumbnails are the following:

- WANTS MORE gun restrictions
- WANTS FEWER gun restrictions
- Not enough information

Each participant was shown 20 thumbnails (5 pages with 4 randomly-selected thumbnails on each page). Due to a glitch, three participants saw more than 20 video thumbnails (one person saw 22 and two people saw 24). On average, each video received **83.68** ratings.

D. Participant Recruitment and Compensation. We recruited 999 participants (out of a targeted 1000) from Amazon.com's Mechanical Turk (via CloudResearch). Participants were assigned either to view the minimum wage or the gun control videos. After removing participants with incomplete or duplicate data, a total of **966** unique participants completed the experiment (637 for the minimum wage videos and 329 for the gun control videos). In post-hoc analysis, we identified a further 7 individuals who had suspicious voting behaviors (e.g., "straight-lined" their responses by always answering the same thing, giving inconsistent responses when repeatedly shown the same thumbnail); however, their responses do not affect our overall results.

Participants were compensated at a base rate of \$0.50. To incentivize attentiveness, they were also be paid an accuracy bonus of \$0.05 per thumbnail if they were able to correctly identify its ground-truth label. Since the task lasted 3–4 minutes in total and involved labeling 20 thumbnails, a randomly-guessing participant could expect $\$0.50 + \$0.05 \times 10 = \$1$, roughly a \$15 per hour base payment rate, with the possibility of earning up to \$1.50 or roughly \$22.50 per hour.

E. GPT-4V Baseline. We also compare human annotations to a computational baseline, in which we provide GPT-4V with the same information as the human raters and ask it to annotate the video thumbnails. As this used a state-of-the-art language and vision model, we regard this baseline as approaching the limit of accuracy when using the recommendation page information alone (i.e., the thumbnail, title, and channel name) to infer information about a video's partisanship. The prompt used for GPT-4V is provided in Section 19, Heading A.

F. Results. We are interested in two areas of outcomes: (1) The *individual-level performance* (that is, for a given participant, how many thumbnails are they able to guess correctly out of 20?) and (2) The *video-level performance* (that is, among all videos, what percentage can be guessed correctly?). For the former, we examine the 20 videos shown to an individual, comparing their ratings to the ground truth; for the latter, we aggregate all ratings for a video by majority vote, then compare these to the "ground truth." We note that, for 21 videos (9.375%), the majority vote was "not enough information;" therefore, at the video level, accuracy is strictly deflated (since, by definition, "not enough information" does not match the ground truth label); we therefore present an exploratory analysis in which we remove votes for "not enough information" and take the label with the next largest share of votes (see Table S4).

F.1. Individual-Level Performance. Overall, we find that individuals correctly identify a video's partisanship (Liberal/Conservative) 56% of the time, a value statistically significantly different from random guessing ($t = 10.946, p < 0.01$). However, in an exploratory analysis, we observe that there is heterogeneity across different topics and partisan leanings; for example, while accuracy for all other topic categories ranges from 61-63%, individuals struggle with identifying the partisanship of Liberal Gun Control videos, with an accuracy of just 48%—on par with guessing ($t = -1.737, p = 0.083$)[‖].

F.2. Video-Level Performance. Collectively, the crowdsourced "majority vote" labels correctly identify a video's partisanship 71% of the time, which is also statistically significantly different from random guessing ($t = 6.726, p < 0.01$). When votes for "not enough information" are removed, this value further increases to 76%.

We further observe that randomly sampled individuals are significantly less accurate than the GPT-4V baseline in identifying the partisanship of a video ($t = -4.267, p < 0.01$). The high rates of accuracy in the GPT-4V baseline suggest that there is meaningful signal in the "first impression" information on the recommendation page to indicate a video's partisanship, suggesting that perhaps with higher accuracy bonuses, better training, or expert coders, there may be potential for improving human raters' accuracy.

Similar to the individual-level results, however, we observe heterogeneity in accuracy across different topics and partisan leanings; in general, partisanship for minimum wage videos appeared to be easier to discern than partisanship for gun control videos (76% for human annotators and 91% for GPT-4V; compared to 60% for human annotators and 69% for GPT-4V), and partisanship for conservative videos appeared to be easier to discern (78% for human annotators and a surprising 93% for GPT-4V) than partisanship for liberal videos (compared to 67% for human annotators and 79% for GPT-4V).

[‖] Here, we operationalize guessing conservatively as a 50-50 random chance, even though, in reality, participants had a third option — choosing "Not enough information." If we were to operationalize guessing as a 1 in 3 chance, the value of 48% is significantly better than chance ($p < 0.01$)

	Humans<br>N = 224 videos	Humans (Dropping "Not Enough Info" Votes)<br>N = 224 videos	GPT-4V<br>N = 222 videos
**Overall Accuracy**			
Global	0.71	0.76	0.84
Minimum Wage	0.76	0.82	0.91
Gun Control	0.60	0.64	0.69
**Accuracy on Liberal Videos**			
Global	0.67	0.71	0.79
Minimum Wage	0.73	0.77	0.83
Gun Control	0.58	0.62	0.73
**Accuracy on Conservative Videos**			
Global	0.78	0.86	0.93
Minimum Wage	0.78	0.87	0.98
Gun Control	0.77	0.82	0.77

Table S4. Accuracy comparison between humans and the GPT-4V baseline. The first column shows accuracy metrics by treating the majority vote as the label (the analysis stated in the pre-analysis plan); the second column presents an exploratory analysis in which votes for "not enough information" are removed, and the label with the next most votes is treated as the majority label.

G. Exploration of Content Perception Accuracy for Different Subsets of Videos. We next explored participants' accuracy in discerning the ideological orientation of different video subsets. We first weighted videos by the number of times participants chose to view them in Studies 1–3. This analysis allows us to understand whether participants were better at perceiving the ideological leaning of videos that were actually chosen. We then explore perception accuracy in the subset of videos that had the the most unambiguous ideological content.

G.1. Weighted Analysis by Number of Views. To measure the number of times participants *chose* to watch a video when it was recommended to them, we compiled the number of times each video was viewed across Studies 1–3, removing "seed" videos (which were presented to participants without choice). We then applied a weighted average, in which the accuracy of perceiving the ideological content of a video was computed as follows. Let v_i represent the number of times a given video i was viewed, and let a_i represent the binary accuracy of whether participants were able to assess the ideological leaning of a given video i from its first-impression information. The weighted average is then $v_i a_i / \sum_i v_i$.

Since participants had access to different sets of recommendations at each level of the recommendation tree, we conduct the analysis separately for each level (counting only the views of a video at a given level of the tree), as well as pooled across all levels. We also separately analyze the subset of Liberal and Conservative videos. Our results are presented in Table S5.

	All Videos	Liberal Videos	Conservative Videos
Level 1	0.667	0.506	0.832
Level 2	0.614	0.408	0.836
Level 3	0.643	0.512	0.788
Level 4	0.654	0.561	0.782
Pooled (All Levels)	0.644	0.493	0.813

Table S5. Accuracy weighted by the number of times participants chose to view the videos in Studies 1–3.

We observe that, in general, the weighted accuracy of perceiving a video's ideological content is slightly lower than the unweighted accuracy presented in Table S4 (64%, compared to 71%). However, this difference appears to be entirely driven by a lower accuracy in perceiving the ideology of liberal videos from their first-impression information — participants have only a 49% weighted accuracy in perceiving the ideology of a liberal video (far lower than its unweighted average of 67%), compared to an 81% weighted accuracy of perceiving the ideology of a conservative video (higher than its unweighted average of 78%). These results are consistent with our earlier finding that conservative videos are, in general, easier to identify from their recommendation page information.

We speculate that this heterogeneity in perceiving liberal versus conservative content may be due to differences in how liberal and conservative videos tend to present information—a manual inspection of some of the top-viewed videos shows that, while conservative videos tend to make their ideology very clearly from the outset ("Ben Shapiro Kills the Minimum Wage Argument for Good"), liberal videos tend to take a neutral or ambiguous stance ("Fast Food CEO After Minimum Wage Increase: 'I was stunned by the business"'); Figure S11.

G.2. Subset Analysis for Unambiguously Ideological Minimum Wage Videos. We next conduct a subset analysis in which we examine a subset of 107 minimum wage videos for which the human "gold-standard" ratings using the full video content, GPT-4V ratings of ideological extremity using first-impression content and a video transcript, and a BERT measure from(6) all agreed on a video's ideology. We take this subset to be videos whose content can be unambiguously judged to be partisan regardless of

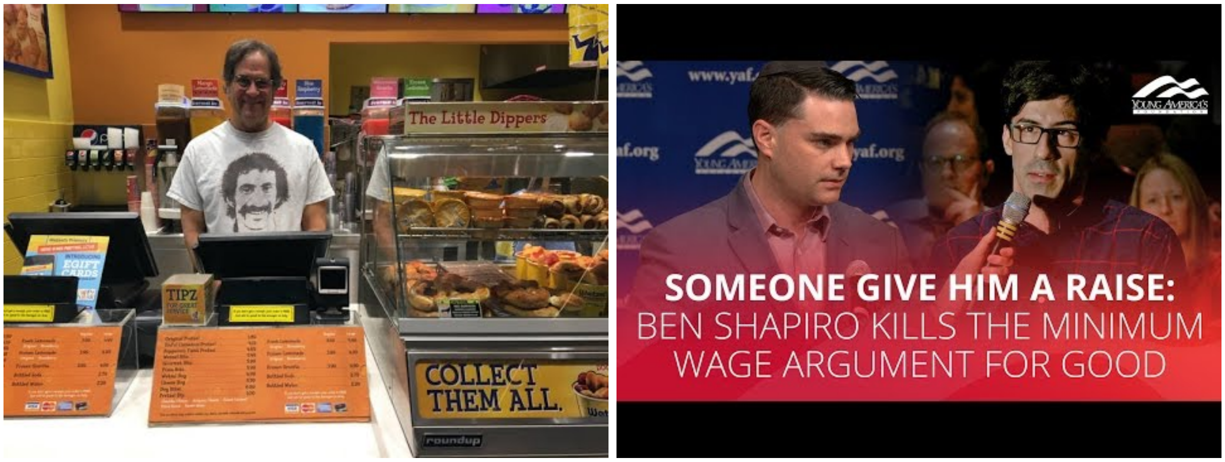

Fig. S11. Example of a Liberal versus Conservative Thumbnail. The above two thumbnails represents one of the top-viewed liberal (left) and conservative (right) videos, respectively. The liberal video, “Fast Food CEO After Minimum Wage Increase: ‘I was stunned by the business’,” had 1024 views, and the conservative video, “SOMEONE GIVE HIM A RAISE: Ben Shapiro kills the minimum wage argument for good,” had 934 views. Notably, the conservative video presents much clearer cues about its ideology: it shows a well-known conservative commentator (Ben Shapiro), and it explicitly denigrates the minimum wage (“kills the minimum wage argument”). In contrast, the liberal video presents ambiguous content; the thumbnail image is a man at a cash register, and the title of the video makes a neutral statement (that the CEO is “stunned”), without explicitly supporting the minimum wage.

the human or algorithmic method. (For more details on the process of rating a video’s political extremity, please see B.1; we further comment on the identification of “ambiguous” videos in B.2.)

Within this “unambiguous” subset, we find that the overall human accuracy is 0.77; accuracy among liberal videos is 0.76; and accuracy among conservative videos is 0.91. While these numbers are all higher than their counterparts on the full set of videos (Table S4), we note again that the accuracy increase is substantially higher for conservative videos in particular — reinforcing the earlier observation that conservative videos may tend to make rhetorical choices that strongly convey their ideology to viewers at a quick glance.

H. First-Impression Labeling: Summary. Taken together, the results of the First-Impression Labeling experiment demonstrates that participants are generally aware of the ideological leaning of a video from the recommendation page information—thus, when presented with a series of choices, they have information about what they are choosing to watch. While the information communicated through a video thumbnail is noisy, imperfect, and heterogeneous depending on the topic and ideological leaning (with conservative videos being much more clear in conveying ideological signals than liberal videos), when making decisions about what to watch next, we believe that it is safe to assume that the recommendation page conveys important information about a video’s ideology, serving as a manipulation check for our studies.

14. "Rabbit Hole" Experiment (Study 4)

A. Motivation. This is a one-wave study that closely mirrors the design of the original Minimum Wage experiments (Studies 2 and 3; Preregistration). Relative to the original Minimum Wage experiments, this study makes two changes to the experimental procedure. First, the collection of pre-treatment characteristics takes place immediately before the treatment, rather than a separate "wave" in the prior week. Second, rather than providing participants with recommendations and allowing them to choose the next video to watch, we remove the element of choice, mimicking the behavior of the YouTube Shorts platform. Participants instead are randomly assigned to deterministic sequences of either constant or increasing extremity, in which the subsequent video plays automatically after the prior video completes.

In this design, we deviate from the approach of Studies 1–3, which operationalized political ideology in a binary manner as either liberal or conservative. These studies did not attempt to distinguish between "filter bubbles" that slanted recommendations toward a specific ideological leaning and "rabbit holes" that also increase the extremity of their ideological positions over time. It is possible, for example, that participants in Studies 1–3 were consistently exposed to ideological videos of a similar level of ideological extremity.

In Study 4, we conduct a more explicit test of the "rabbit hole" hypothesis that viewers are polarized by platform decisions that push them into watch sequences of increasing extremity. To do so, we operationalize the ideological position of a video as a continuous variable, and we test whether viewing increasing-extremity sequences changes our main policy-attitude outcome. Specifically, by using the GPT-4V continuous measure of ideological "extremity"—along with extensive manual review and curation by authors—we are able to curate sequences of five videos that either grow in ideological extremity over time ("increasing") or remain at a roughly constant level of ideological extremity ("constant"). We then randomize participants into viewing either "increasing" or a "constant" sequences. Unlike in the original experiment, Study 4 removes the element of choice, so that participants cannot select the next video in a given sequence (though they can choose to skip ahead to the next video, much like on the YouTube Shorts platform). This design decision was necessary given the amount of author labor required to manually review, reorder, or substitute videos to ensure that over-time ideological extremity of a candidate sequence fully captured the desired patterns.

In summary, Study 4 builds upon Studies 1–3 to assess whether conclusions differ when explicitly manipulating the algorithm to produce "rabbit holes" of increasing extremity.

B. Curation of Increasing and Constant Sequences. We tested numerous approaches for measuring the extremity of the content and ultimately determined that GPT annotations of political extremity—based on full transcript, channel name, and thumbnail image—appear to perform best when compared with human annotations. Specifically, we utilized OpenAI's GPT-4V, which can incorporate visual information from a video's preview thumbnail, which can often be informative. We evaluated a number of other approaches from recent work, including Lai et. al.'s (6) pretrained model using title/description metadata and Hosseinmardi et al. (7) expert classification of the channel/creator extremity. However, we found that these approaches performed poorly in recovering our own human labels, and we ultimately concluded that it was essential to incorporate the actual transcript of arguments made in the video.

B.1. Continuous Extremity Rating. To generate sequences that either increased or remained constant in their extremity, we transcribed all videos in the minimum wage dataset using the Whisper API by OpenAI. We then provided the video transcript, thumbnail, and channel name to GPT-4V ("gpt-4-vision-preview") with a prompt (Section 19, Heading B), in which we asked GPT-4V to provide a rating between –1 (extremely liberal) and +1 (extremely conservative) on the video's political leaning. Due to a race condition in the code parallelization, some videos received multiple ratings from GPT-4V, which had slight variation between repeated queries; in cases of repeated ratings, we take the average of all ratings for a given video ID. We also demonstrate the robustness of all findings against other means of aggregation (e.g., randomly sampling one rating among videos with multiple ratings).

To check that GPT-4V ratings match the "ground truth," which is the original binary hand-labelings of whether a video was liberal or conservative, we examined whether the sign of the GPT-4V ratings (negative if liberal, positive if conservative) matched the original binary labels. Not excluding missing ratings (which occurred either when the video had been removed from YouTube, and hence could not be rated, or if GPT-4V refused to rate a video due to a content safety violation) GPT-4V achieved a 85.7% match with the original binary labels. Excluding missing ratings, it achieved an 87.8% match with the original labels.

We further compared the continuous ratings from GPT-4V with two other established systems for quantifying the political extremity of YouTube videos: a pretrained BERT model using video metadata by (6), and a channel-based extremity rating by (8). Figure S12 demonstrates that the GPT-4V rating out-performs that of BERT. Figure S13 demonstrates that the GPT-4V rating appears to be qualitatively correct for partisan channels, and it offers an improvement over channel-level labels for centrist channels. While the channel labels used by (8) cannot distinguish between a left-leaning video on a centrist channel and a right-leaning video on a centrist channel, our continuous measure is able to effectively distinguish between them.

B.2. Imperfections in "Ground Truth". One consequence of this ideological-extremity analysis, which was suggested by a reviewer, is that in a small number of cases it led to manual review revealing imperfections in the human annotations that we treated as the "gold-standard" labels. We identified 7 cases in which both GPT-4V and the BERT method from (6) agreed on an ideology, but disagreed with previous human "gold-standard" labels. We present these cases in more detail in Table S6, concluding that

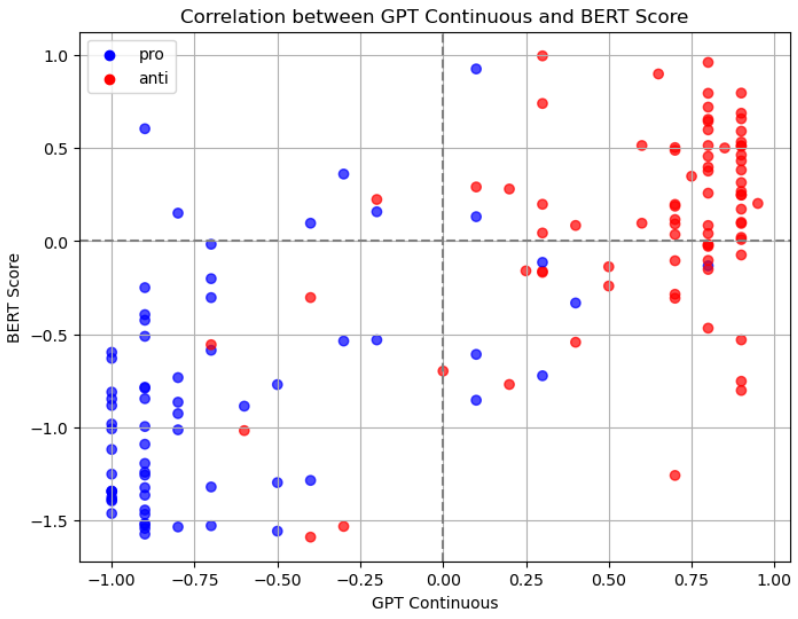

Fig. S12. Comparison between the continuous GPT-4V ratings and ratings from the pretrained BERT model from (6). Red dots represent videos in which the "gold standard" label was conservative (anti-minimum wage), while blue dots represent videos in which the "gold standard" label was liberal (pro-minimum wage). The x-axis represents the continuous rating from GPT-4V, and the y-axis represents the continuous rating from (6). Red dots in the left half of the graph represent misclassification by GPT-4V, in which the true label is conservative, but GPT-4V assigned it a liberal score; blue dots in the right half of the graph represent misclassification by GPT-4V, in which the true label is liberal, but GPT-4V assigned it a conservative score; blue dots in the top half of the graph represent misclassification by BERT, in which the true label is liberal, but BERT assigned it a conservative score; red dots in the bottom half of the graph represent misclassification by BERT, in which the true label is conservative, but BERT assigned it a liberal score. Overall, GPT-4V has a substantially lower error rate than BERT. Note that a later review described in SI B.2 indicated that a small number of "gold standard" liberal or conservative labels, despite being based on the consensus of human coders using the full video, were incorrectly applied to ambiguous videos in a way that may inflate error rates.

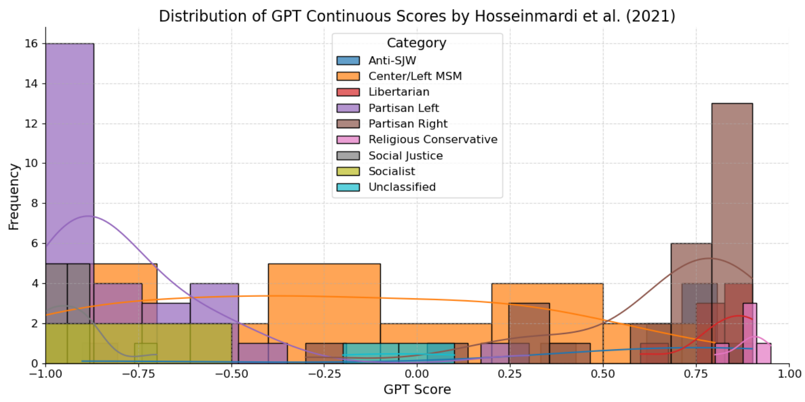

Fig. S13. Comparison between the continuous GPT-4V ratings and channel labels from (8). Overall, the continuous GPT-4V measure assigns liberal scores to "Partisan Left" videos and conservative sores to "Partisan Right" videos, which qualitatively validates our measure. However, categorical labels are unable to differentiate between centrist channels (e.g., "Center/Left MSM"), while our continuous variables can effectively draw a distinction between liberal and conservative videos sourced from centrist channels.

such cases are ambiguous (often presenting information from both sides). However, they represent a worst-case error rate in the "gold" labels that is less than 4%.

Overall, given extremely high agreement with the original human labels (over 85%), and given that a portion of these mismatches represent errors in the original labels rather than the GPT-4V extremity measure, that the continuous measure represents a reasonable operationalization of a video's ideological extremity.

B.3. Curation of Increasing and Constant Sequences. Having established a method of obtaining a continuous measure of political extremity, we then curate a set of "increasing" and "constant" video sequences in a two-stage process.

In Stage 1, we use the ratings from GPT-4V to sample sets of five videos that either span a range of levels of extremity (thus

Video ID	GPT-4V and BERT Label	Original Gold Label	Description	Comment
-acK4XLbjL0	Liberal	Conservative	Video from a centrist outlet (The Hill) in which a Biden spokesperson states that the President is in support of a $15 minimum wage, but commentators point out that not enough Democrats support the measure.	Gold Incorrect
6OTWpLU0_qU	Liberal	Conservative	Video from a strong progressive talk show host (Thom Hartmann) in which he debates a conservative.	Gold Incorrect
AlMUIkgvazo	Conservative	Liberal	Video from a conservative outlet (Fox News) in which the host is against the wage, but a Democrat being interviewed argues in favor of the minimum wage.	Gold Incorrect
CaE21Qhjgr0	Liberal	Conservative	Video from a centrist outlet (The Hill) in which the hosts argue that Sinema is being politically punished by constituents for her opposition to the minimum wage.	Gold Incorrect
WSDnRbxGlFw	Conservative	Liberal	Video arguing that fears of automation are not a reason for not raising the minimum wage.	Gold Correct
Z_r5TlBdjEM	Liberal	Conservative	Video making arguments for both sides: it points out that the wage has been stagnant for a long time and is no longer livable, but also cites counterarguments (burdens on small businesses; automation; unemployment). Seems to lean slightly liberal.	Gold Incorrect
v8bnRfvMVMg	Liberal	Conservative	Second video from a strong progressive talk show host (Thom Hartmann) in which he debates a conservative.	Gold Incorrect

Table S6. Minimum Wage Videos for which BERT and GPT-4V Agreed, but Disagreed with the Gold Standard Labels. We identified 7 videos for which BERT and GPT-4V were in alignment, and both disagreed with the human labels that we treated as the gold standard. A manual inspection of each of these videos finds that many are ambiguous because they either present information from both sides of the minimum wage debate, or involve a conversation between two people (one liberal, one conservative). In cases of such conversations, our instruction had been to treat the video's ideology as that of the host; thus, by this definition, several of the human labels were incorrect. However, the number of incorrect gold labels represents a very small number of the total number of minimum wage videos (6 out of 154, or less than 4%).

ensuring that there is a sense of "increasingly" intense ideology), or that are all from a limited, moderate level of extremity (thus keeping the level of ideological extremity "constant"). We apply the following method:

- We first filter all videos to those with the same "ground truth" label. That is, for a liberal sequence, we select only videos that have a liberal (pro-minimum wage) ground truth label; for a conservative sequence, we select only videos that have a conservative (anti-minimum wage) ground truth label.

- For "increasing extremity" sequences, we sample five videos whose absolute ratings fall in three value ranges: 0-0.5 ("moderate"; 2 videos); 0.5-0.7 ("intermediate"; 2 videos); and ≥ 0.7 ("extreme"; 1 video). For "constant" sequences, we sample all five videos from the "moderate" value range.

- For "increasing extremity" sequences, we initially ordered the videos based on their rating, from lowest to highest absolute value. For "constant" sequences, we randomized the order of the videos.

In Stage 2, two authors watched the full video sequences and verified that the sequence created a qualitative sense of increasing extremity. In addition, authors made the following ad-hoc adjustments to improve the stimuli:

- **Swaps**: Authors sometimes changed the order of videos in a sequence if some videos felt more or less qualitatively extreme than their GPT-4V labels suggested. For example, videos with higher ratings may not have felt as qualitatively extreme because the way in which the argument was conveyed was in a dry and academic manner, while videos that were given a lower rating may have felt more extreme because of the way the video expressed its argument. These manual corrections were necessary because GPT-4V did not have access to the full multi-modal video (e.g., the tone, expression, and other subtle elements that create a subjective feeling of extremity).

- **Replacements**: Some videos were randomly selected with higher frequency than others. If any video appeared more than 3 times within a given treatment arm, it was replaced with another video of comparable orientation and extremity.

We generated a total of 24 unique sequences, with six sequences each for four treatment arms: (1) liberal and increasing extremity; (2) liberal and constant extremity; (3) conservative and increasing extremity; and (4) conservative and constant extremity. The sequences used in the study, along with documented rationales for swaps and replacements, are presented in this spreadsheet.

C. Infrastructure. In the experiment, we use a simplified version of our YouTube-like experimentation platform, which serves fixed sequences of videos to users. Figure S14 depicts the interface.

Our design was inspired by the YouTube Shorts interface, in which participants have access to a simplified version of the platform. In YouTube Shorts, individuals are unable to select or rewind videos; they can only choose to watch the current video or skip ahead to the next video. Our design is also similar to the "autoplay" YouTube feature, which automatically selects the top recommended video after the current video concludes or a user skips forward.

D. Implementation Details.

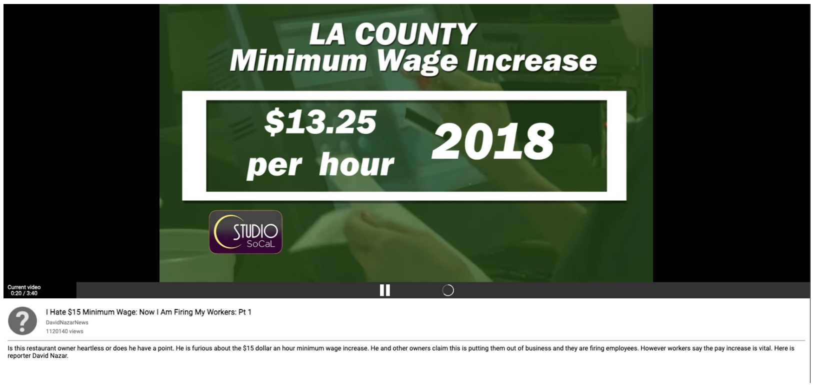

Fig. S14. Video Watching Interface. The Study 4 interface mimics YouTube and is based on the original platform used in Studies 1–3. Recommendation pages between videos are removed, and participants can only move forward to the next video in the sequence after the minimum watch time expires. In addition, thumbs-up, thumbs-down, save, and rewind options are removed. The video must be watched for at least 30 seconds, after which the viewer can click the skip button to jump to the next video.

D.1. Participant Recruitment. We recruited 1032 participants (based on a target of 1,000 completes) from Amazon.com's Mechanical Turk (via CloudResearch). After removing participants who failed the attention check questions, 932 participants remained. We further excluded three subjects who had null interface duration times from the study, and conducted our statistical analysis with the remaining **929** subjects.

D.2. Treatment Arms. We randomly assigned respondents to a sequence type ("increasing" or "constant"), but to account for heterogeneous treatment effects among ideologically extreme individuals, the ideological leaning of the video sequence depended on the person's pre-treatment policy attitudes.

Using tercile cutoffs from a 250-person pilot sample, we calculated a pre-treatment minimum wage policy index for each participant and labeled individuals "Liberal," "Moderate," or "Conservative." Those in the lower tercile were assigned a "Liberal" label; those in the middle tercile were assigned a "Moderate" label; and those in the top tercile were assigned a "Conservative" label. We then retained these cutoffs for the full Study 4 sample, targeting 1,000 participants.

The distribution of respondent pre-treatment attitudes differed slightly in the full study. Based on cutoffs established from the pilot sample, we ultimately assigned 41% of the participants to the liberal condition (379 subjects), 32% to the moderate condition (296 subjects), and 27% to the conservative condition (257 subjects).

As in Studies 1–3, because people generally seek out pro-attitudinal videos in real-world YouTube usage, we did not assign respondents to counter-attitudinal seed videos. Instead, we block-randomized treatments such that "moderates" were assigned a sequence of videos with random ideological orientation (either in favor or opposed to raising the minimum wage), while "liberals" were only assigned sequences that were in favor of raising it and "conservatives" were only assigned sequences that were opposed.

In summary, we have four treatment arms (with the number and types of individuals assigned to each condition indicated in parentheses):

1. Pro-increasing minimum wage, increasing extremity (186 liberals, 75 moderates);
2. Pro-increasing minimum wage, constant extremity (193 liberals, 74 moderates);
3. Anti-increasing minimum wage, increasing extremity (110 conservatives, 71 moderates);
4. Anti-increasing minimum Wage, constant extremity (147 conservatives, 76 moderates)

Within each treatment arm, participants are randomly assigned to watch one of the six curated sequences (as described in Section B.3).

E. Analysis.

E.1. Primary Analysis. The first set of hypothesis tests is analogous to that of Studies 1–3, except that we focus on only a single outcome, the policy-attitude index. We test for effects in the following contrasts:

1. Increasing- vs. constant-extremity assignment among liberal participants;
2. Increasing- vs. constant-extremity assignment among conservative participants;
3. Increasing- vs. constant-extremity assignment among moderate participants assigned to a liberal sequence;
4. Increasing- vs. constant-extremity assignment among moderate participants assigned to an conservative sequence;
5. Liberal vs. conservative video orientation among moderate participants with an increasing-extremity algorithm; and
6. Liberal vs. conservative video orientation among moderate participants with a constant-extremity algorithm.

As noted above, the outcome is the post-treatment minimum wage policy index, and the sole control variable is the pre-treatment minimum wage policy index.

We thus test six hypotheses. The first four four relate to whether the randomized manipulation of the algorithm (increasing or constant extremity) has any discernible overall effect on policy attitudes as measured by the minimum wage attitude index. The latter two relate to randomized manipulation of the ideological orientation of content to which respondents are exposed. All contrasts are made within the predefined liberal, moderate, and conservative subgroups. To control the false discovery rate in the presence of multiple testing, we apply the Benjamini-Hochberg correction (5).

E.2. Omnibus Linear Test. In an effort to ensure that null estimates in the first four hypotheses (effects of algorithmic interventions) were not due to a lack of power, we then conducted a second analysis that pooled across groups of respondents. To facilitate this, we reverse-coded the outcome among participants who were exposed to the liberal seed, as this ensures that a positive change in the recoded outcome means a shift in the policy direction espoused by the videos.

We then ran a simple regression of the *difference* in pre- and post-treatment policy beliefs (the wage index) on a binary indicator for whether respondents had been assigned to an increasing- or constant-extremity sequence. This tests the null hypothesis that the nature of the algorithm has no effect on the individual's policy position.

F. Results.

F.1. Omnibus Test. Table S7 shows the omnibus test conclusion. We found weakly suggestive evidence ($p = 0.069$) for a possible increasing-vs.-constant-extremity algorithmic effect when pooling across respondent types (i.e., comparing increasing vs. constant sequences of the same ideology shown to the same type of respondent, but estimating a single treatment-effect coefficient).

	**Dependent Variable**
	**Difference between post and pre opinions**
**Policy Index Difference**	0.037* (0.021)
**Constant**	-0.017 (0.015)
**Observations**	929
**Adjusted R^2**	0.0025
Note:	*p<0.1; **p<0.05; ***p<0.01

Table S7. Omnibus Test Results

15. Assessment of Learning Effects

We examined the extent to which participants felt that they had learned new ideas or arguments through watching the video sequences, and we evaluated whether treatment assignment discernibly had effects on learning.

The 929 participants in the "Rabbit Hole" experiment (Study 4) who completed the study and passed attention checks were asked a yes/no question about whether they learned anything through watching the videos. 926 of the participants also responded to an optional open-response question about what they learned (answering "yes" the former was not a precondition for responding to the latter).

As a check on the validity of yes/no responses, we used GPT-4 to classify whether the open-ended response indicated that the participant had learned anything. We compared the self-reported binary question with the resulting text classification. The prompt used for GPT-4 is provided in Section 19, Heading C. Those who did not provide an open response were assumed to not have learned anything.

Across both outcome definitions, **89%** of participants reported that they had learned something through watching the videos. However, responses did not perfectly correspond: the two methods matched 91% of the time. For example, a small number of individuals described themselves as not learning anything new, while nevertheless writing about something they had learned in their open response.

	Yes	No
**Binary Question**<br>Did you learn anything about the minimum wage debate?	828 (89.1%)	101 (10.9%)
**Open-Ended Response**<br>What did you learn about the minimum wage debate?	825 (88.8%)	104 (11.2%)

Table S8. Learning from Minimum Wage Videos.

A. Learning by Initial Partisanship. In general, moderates and liberals tended to self-report learning the most. Among moderates, 91.5% responded "yes," and GPT-4 characterized 90.2% of them as providing text indicative of learning. Among liberals, 89.9% responded "yes" and 91.8% of text responses indicated learning. The least amount of learning took place among conservatives, with 85.2% responding "yes" and 82.8% of text responses indicating learning.

B. Learning by Treatment Assignment. We find consistent results across both the self-reported (Table S9) and GPT-4 classification of open-ended responses (Table S10) measures of learning. Results are organized as follows. The "LL subset" column analyzes only liberal respondents, all of whom were assigned to liberal content per the design described above. The "ML" and "MC" columns analyze the subsets of moderates randomized into watching liberal and conservative content, respectively. Finally, the "CC" column analyzes conservative respondents, all of whom were assigned to conservative content. Within each column, a baseline coefficient represents the average learning rate under the constant-extremity algorithm, and the "increasing extremity" coefficient represents the difference between the treatment arms. Finally, the "pooled" column reports the result of a regression in which fixed effects are estimated for each of the preceding groups, with a single treatment effect estimate that pools over all groups.

Results can be summarized as follows: across all regressions and subgroups, we find no treatment effect of watching an extreme video sequence on learning new information about the video. Differences in learning appear to primarily occur by partisan subgroup; liberals may report learning more than conservatives ($t = 1.61$ $p = 0.108$ for binary measure; $t = 3.05$ $p = 0.002$ for open-response measure) as do moderates who are assigned to liberal content ($t = 2.31$ $p = 0.021$ for binary measure; $t = 2.54$ $p = 0.011$ for open-response measure).

C. Qualitative Analysis of Open-Ended Responses. We performed a qualitative analysis of the 926 open-ended responses, aided by BERTopic, on the open-ended responses. Specifically, we used a BERT-based topic model with the HDBSCAN clustering method (with minimum cluster size of 20 documents), and a UMAP-based dimensionality reduction with 3 neighbors and 10 components. The analysis yielded 24 topics, which an author then manually read, grouped, and classified into human-understandable categories of learning.

We next present a collection of representative responses across the topics. While we emphasize that this analysis is purely exploratory, the open-ended responses shed light on the insights that stuck with participants after they watched the videos. These included learning about the negative implications of the minimum wage on small-business profitability and survival; learning that some business leaders support increased wages; learning about potential negative implications for job losses and inflation, and so on. Having reviewed an exceptionally large amount of minimum-wage content of varying extremity, we qualitatively recognized these arguments as having been made by both moderate and extreme videos, suggesting that "extremity" can manifest in ways other than bringing up new arguments.

C.1. General Knowledge or Adding Nuance. *Participants reported learning more about the minimum wage debate in general, and adding nuance to their perspectives even when they had some initial knowledge.*

- I didn't know much about it before so I learned a lot. I learned the reasons why people oppose it and I also learned reasons why their issues with it aren't as significant as it's made out to be and also how increasing the minimum wage can benefit the entire economy.

	Dependent Variable: Self-Reported Binary Response				
	(LL subset)	(ML subset)	(MC subset)	(CC subset)	(Pooled)
Increasing Extremity	-0.007	-0.001	-0.005	0.045	0.008
	(0.031)	(0.041)	(0.051)	(0.045)	(0.020)
Liberal Respondents, Liberal Content	0.903***				0.895***
	(0.022)				(0.019)
Moderate Respondents, Liberal Content		0.933***			0.929***
		(0.029)			(0.027)
Moderate Respondents, Conservative Content			0.900***		0.893***
			(0.037)		(0.028)
Conservative Respondents, Conservative Content				0.826***	0.847***
				(0.034)	(0.023)
Observations	378	149	146	256	929
R^2	0.000	0.000	0.000	0.004	0.008
Adjusted R^2	-0.003	-0.007	-0.007	0.000	0.004
Residual Std. Error	0.301 (df=376)	0.252 (df=147)	0.306 (df=144)	0.356 (df=254)	0.311 (df=924)
F Statistic	0.057 (df=1; 376)	0.000 (df=1; 147)	0.011 (df=1; 144)	1.001 (df=1; 254)	1.831 (df=4; 924)
Note:					*p<0.1; **p<0.05; ***p<0.01

Table S9. Regression analyses of learning, based on yes/no self-reports. "Increasing Extremity" coefficients represent the estimated effect of an this algorithmic intervention relative to a constant-extremity baseline. Subsequent coefficients represent baseline outcome means among the constant-extremity group. LL, ML, MC, and CC analyses analyze the corresponding subsets of respondents; the pooled analysis analyzes all four groups together with a single treatment-effect coefficient.

	Dependent Variable: Open-Ended Response Classified by GPT-4				
	(LL subset)	(ML subset)	(MC subset)	(CC subset)	(Pooled)
Increasing Extremity	-0.013	-0.001	0.051	0.052	0.017
	(0.028)	(0.045)	(0.053)	(0.048)	(0.021)
Liberal Respondents, Liberal Content	0.925***				0.910***
	(0.020)				(0.019)
Moderate Respondents, Liberal Content		0.920***			0.911***
		(0.032)			(0.028)
Moderate Respondents, Conservative Content			0.857***		0.875***
			(0.038)		(0.028)
Conservative Respondents, Conservative Content				0.798***	0.819***
				(0.036)	(0.023)
Observations	378	149	146	256	929
R^2	0.001	0.000	0.006	0.005	0.016
Adjusted R^2	-0.002	-0.007	-0.001	0.001	0.012
Residual Std. Error	0.275 (df=376)	0.274 (df=147)	0.322 (df=144)	0.378 (df=254)	0.314 (df=924)
F Statistic	0.220 (df=1; 376)	0.001 (df=1; 147)	0.905 (df=1; 144)	1.193 (df=1; 254)	3.740*** (df=4; 924)
Note:					*p<0.1; **p<0.05; ***p<0.01

Table S10. Regression analyses of learning, based on GPT-4 coding of open-ended responses. "Increasing Extremity" coefficients represent the estimated effect of an this algorithmic intervention relative to a constant-extremity baseline. Subsequent coefficients represent baseline outcome means among the constant-extremity group. LL, ML, MC, and CC analyses analyze the corresponding subsets of respondents; the pooled analysis analyzes all four groups together with a single treatment-effect coefficient.

- I found it to be a more nuanced argument than initially expected. I thought it was well represented that minimum wage and how we think about it depends on personal viewpoints. Some view it as a question of equity and that by increasing the wage we are giving a more equitable outcome to employees who then will spend their increased purchasing power to help the economy. Some view it as a question of economic principles, that by awarding a higher minimum wage, we are awarding dominant firms because they can eat the cost associated with a higher minimum wage while increasing the barrier to entry for new competition. Overall, some good and thoughtful discussion between the five videos.
- There are two sides and that both sides have valid points for raising and or maintaining that minimum wage. I also learned that some companies have gone ahead and raised the minimum wage before it is required. For instance, Amazon has already raised their minimum wage to $15.00 an hour.
- It is very much a partisan issue (which I already knew), but there are also other variables to consider such as the fact that different states have different costs of living. A nationwide universal standard minimum wage could put a greater burden on employers in states where there is a lower cost of living and could short change workers in states where there is a higher cost of living.

C.2. Impact to Businesses. *Participants reported being surprised at the implications of the minimum wage on businesses (particularly smaller businesses and restaurants).*

- I had no idea it was such a big deal. I guess I never thought about how many people would lose their jobs or get less hours or have no job security because owners are figuring out ways to cut costs since the wage is increasing.
- It is possible that it hurts small business more than I thought, but I still believe in it. I think if small businesses cannot afford to pay the 15 minimum wage then they should not exist.
- I learned just how difficult it is to expect restaurants to eat the cost of drastically increased labor wages. I learned that the short term gains of a few employees is not worth the longer term losses of a larger number of employees. I also didn't realize that unions were trying to get involved, further exacerbating the issue. I also knew that profit margins in the service industry were pretty thin, but I don't realize how small they were.
- I found the restaurant helper very informative on how a restaurant can use the menu to cut costs and still keep their employees. I also understand how the restaurants are having difficulty keeping up with the sudden increase of minimum wage and that is why they have gone to automation.
- I haven't taken into account the business side of it. I only think about the workers, but also I don't trust businesses to do the right thing so my new information didn't sway me. I wasn't aware [that] to live comfortably you need to make at least [$]16.50 an hour and that's sad knowing the federal minimum wage is so low.

Participants also indicated surprise at the positions of leaders of larger businesses (e.g., Amazon, Wal-Mart, Dunkin, McDonald's).

- I was very shocked to hear that Amazon and Wal-Mart are proponents of a national increase in minimum wage and that their representatives believe that it will actually help small businesses. I knew they had raised rates in response to a lack of workers but I hadn't heard that side of their position before.
- That I think more business owners are opposed than I thought. I hadn't heard Dunkin's CEO speak on this before and it makes me want to go back and earn [sic] more. I think people are split on if it's going to be good for consumers or businesses.
- I was surprised to hear from the McDonald's CEO about the actual steps they're taking to increase wages for all employees. He was surprisingly resilient against the typical complaints people make about wage increases, like having to cut hours, or saying their employees [are] not deserving of a significant wage because they're too young or unskilled. This doesn't make me like the company more, but it's good to see an entity with so much power at least making some kind of argument for increasing wages.

C.3. Impact to Economy. *Participants reported learning about the implications of raising the minimum wage on the broader economy.*

- I learned that it could be even more detrimental to the economy than I thought. It has the potential to decrease the number of jobs available, whilst also making things in the economy MUCH more expensive.

C.4. Impact on Poverty/Affordability. *Participants reported learning about the impact of the minimum wage on individuals' ability to live above the poverty line.*

- That even at [$]15 people are still below the poverty line, that some believe increasing it will slow job growth one important fact when people get more money they will spend all of it those at the poverty level, curious note is inflation is driven by demand and when people spend the extra money demand for products go up increasing their cost and inflation.

C.5. Automation. *Participants reported learning about the role of automation in the minimum wage debate.*

- On[e] thing I had not considered closed was whether rasing [sic] the minimum wage would increase the number of jobs being lost to AI. Both sides weigh in on this, concluding that AI will happen no matter what. Changes in the way we do things over decades has eliminated job[s] but then increased opportunities later, so we would need to see how this would work out.
- I learned that using automation as a reason against raising the minimum wage is not really a valid argument. Because automation will happen regardless if the minimum wage is raised or not.

C.6. Wage Stagnation. *Participants reported learning about the stagnation of "real" wages.*

- I learned that every time [the] minimum wage has went [sic] up, that the economy using inflation has brought it right back down. So in a nutshell, all along minimum wage has stayed the same since the 80's.

C.7. Political Dynamics. *Participants reported learning about the political dynamics of raising the minimum wage, from the mechanics (e.g., the fact that local and federal minimums differ) to the role of different stakeholders (such as unions).*

- California increased theirs to $14 per hour, and Florida recently increased theirs to $15. Both Senator Bernie Sanders and Trump criticized Amazon for lobbying for a minimum wage increase, but then not paying their workers above minimum wage.
- I tended to think that it was all at the federal level without thinking much about it. I learned that States can set a state-wide minimum wage. I learned more details about the line between having too high a wage and less people are hired.

- I learned about the worker union factoring into [the] Democrat motive (although not about how much of that motive). I learned from discussion and comparisons of systems other countries rely on to maintain consistency in the inflation/wage relationship and some subjective observations.
- I wasn't aware that labor unions were pushing for an increase in minimum wage, but asking to be exempt from it.

16. Assessment of Increasingly Extreme Recommendations

We used the extremity scores (which range from –1, extremely liberal, to +1, extremely conservative) to assess the difference between a "current" video's extremity and that of its recommendations. Our dataset consisted of 27,177 recommendations (pairs of current and recommended videos) scraped directly from YouTube's API (described in Section 2), with their associated extremity scores evalued using the method described in Section B.1. We then conducted a regression in which each observation was a current-recommended video pair. Current videos with a greater number of recommendations were downweighted, such that each current video received equal overall weight in the analysis.

We defined the dependent variable as the *difference between current and recommended video scores.* We perform a two-way clustering of standard errors on current and recommended video IDs. Separate regressions were conducted for liberal current videos (with negative extremity scores) and for conservative current videos (with positive extremity scores). The regression in each case assesses the expected difference, over a randomly drawn video from a set (liberal or conservative), between the extremity of a current video and one of its randomly drawn recommendations.

Because the dependent variable is the average change in extremity between the current video and the next recommended video to watch, a positive coefficient on the extremity score would suggest that videos become *more extreme* in the partisan direction of the current video, and a negative coefficient would suggest that a video becomes *more moderate* (i.e., moves away from the partisan direction of the current video, towards zero). Table S11 presents the regression results for each current video category, showing highly significant but substantively small negative coefficients, indicating a slight moderating effect.

	**Current Video Type**	
	**Liberal**	**Conservative**
Intercept	0.0037	-0.0055
	(0.006)	(0.007)
Current Video Extremity Score	-0.0486***	-0.0299***
	(0.006)	(0.008)
**Adjusted R^2**	0.022	0.009
**F-Statistic**	66.85	15.17
**Observations**	13596	13581
Note:	*p<0.1; **p<0.05; ***p<0.01	

Table S11. OLS Regression Results for Assessing Recommendation Extremity Standard errors are robust to two-way clustering by current and recommended video ID.

These results are illustrated in Figure S15),which translates the results onto the scale of the recommendations' extremity score (rather than their difference from the current video). For example, a moderately liberal video with a rating of -0.5 would direct a user to a video with a change of -0.5×-0.0486. Thus, the recommended video would have a expected difference of $+0.0243$ from the current video, or an expected score of $-.4757$, making it slightly more conservative. Similarly, a moderately conservative video with a rating of $+0.5$ would direct a user to a video with a change of 0.5×0.0299, making the next recommended video slightly more liberal than the current video (difference of -0.0150, for an expected extremity of 0.4850).

17. Assessment of Participant Video Selection Against a Random Baseline

In addition to establishing, via the First Impressions Experiment (Section 13), that participants were able to discern the partisanship of a video from only the recommendation page information, we conducted an additional exploratory analysis to confirm that participants in the original Studies 2 and 3 made video choices that significantly differ from randomly watching videos. This analysis enables us to rule out an alternative explanation that participants were simply inattentive or making mindless choices while watching the videos in our study.

A. Analysis Approach. We designed and conducted a test that captures the null hypothesis that respondents are simply conducting a random walk through the recommendation tree. Under this null hypothesis, the fraction of chosen conservative videos is expected to be approximately 61.7%. This is because in the first round, conservative respondents in the slanted condition are offered 3 conserative and 1 liberal recommendation. Under the null, 3 out of 4 participants will choose a conservative recommendation, then receive another recommendation set of 3 conservative/1 liberal video. However, 1 out of 4 participants would randomly choose the liberal recommendation, then receive a 3 liberal/1 conservative recommendation set. Thus, in each round, the expected random-walk conservative choice fraction is

$$\textbf{round 1: } 3/4 = .75$$
$$\textbf{round 2: } .75 * 3/4 + .25 * 3/4 = 0.625$$
$$\textbf{round 3: } .625 * 3/4 + .375 * 3/4 = .5625$$
$$\textbf{round 4: } .5625 * 3/4 + 0.4375 * 3/4 = 0.53125$$

Averaging the four choice rounds yields the overall fraction under the null: 61.7%. (These values were further verified through simulation.) The calculation is simpler in the balanced 2/2 condition, where the expected choice fraction under the null is 50%.

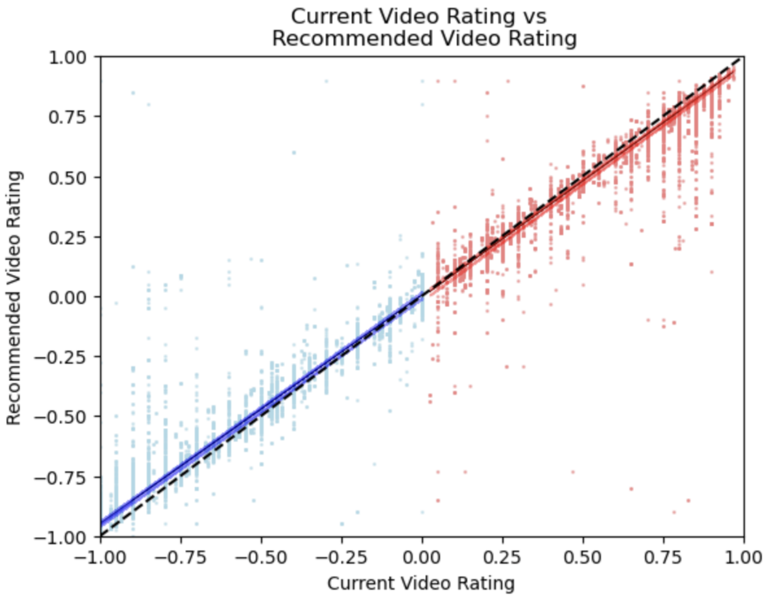

Fig. S15. Relationship Between the Ideological Rating of the Current Video Versus its Next Recommended Video. Here, we show the ideology of the current video (x-axis) against the ideology of its average recommended video (y-axis). More positive values indicate that a video is more conservative (red), while more negative values indicate that a video is more liberal (blue). The dotted line represents parity; a point on the dotted line indicates that a video recommends other videos that have the same level of ideological extremity. In general, liberal videos tend to recommend other liberal videos, while conservative videos tend to recommend other conservative videos. However, extremely liberal points tend to fall above the dotted line, and extremely conservative points tend to fall below the dotted line—indicating a slight "regression to the mean" or moderating tendency in YouTube's recommendations on this topic. In other words, after watching an extreme partisan video, recommendations on the same topic will on average viewers to *less* partisan options.

We probed the random-choice question by reanalyzing interface data from partisan users in the minimum wage studies. We compared the proportion of selected videos that align with the participant's own ideology ("co-ideological") to the expected 61.7% or 50% if the participant were choosing randomly, depending on the level of algorithm slant. We conducted four regressions—(2/2, 3/1) x (liberal, conservative)—on choice level data, clustering standard errors by respondent.

B. Results. We find, across all conditions, that participants are significantly ($p < 0.001$) more likely to select co-ideological videos than expected if they were simply choosing at random.

Across all four statistical tests (liberal/conservative respondents x balanced/slanted algorithm), we reject the null hypothesis that participants are choosing videos randomly:

1. For liberal participants in the balanced condition (n = 2,796 chosen videos), **62.8%** of the chosen videos were liberal, which is significantly different from 50.0% ($p < 0.001$)

2. For liberal participants in the slanted condition (n = 2,808 chosen videos), **66.7%** of the chosen videos were liberal, which is significantly different from 61.7% ($p < 0.001$)

3. For conservative participants in the balanced condition (n = 2,452 chosen videos), **55.1%** of the chosen videos were conservative, which is significantly different from 50.0% ($p < 0.001$)

4. For conservative participants in the slanted condition (n = 2,456 chosen videos), **66.4%** of the chosen videos were conservative, which is significantly different from 61.7% ($p < 0.001$)

We note that respondents who completed fewer than 4 choice rounds were excluded from the primary analysis presented above, due to the complexity of determining the correct overall reference value to test under the null when including them (as these individuals have different individual-level reference values that depend on the number of rounds completed, but are implicitly down-weighted by the regression due to their smaller number of choice observations). It is possible that respondents who do not complete the task are less attentive and could be making choices more randomly. However, including them in the regression does not seem to meaningfully change results (estimates move toward the null by at most one percentage point, when the null is rejected by far larger margins).

Additionally, it is interesting to note that the magnitude of the deviations were markedly smaller for liberal respondents in the slanted arm than liberal respondents in the balanced arm. With a larger recommendation set, this might suggest a saturation point past which providing additional liberal recommendations leads to diminishing returns (e.g. if a certain fraction of liberal respondents are curious about counter-ideological arguments and will always select this option if available).

18. References for Literature Review of PNAS Experiments

To evaluate the length of the stimuli in our study against the typical stimuli length of published experiments, we conducted an extensive review of all media-exposure experiments published in *PNAS* over the past decade that met two criteria: they (1) presented a treatment (e.g., video clips, reading materials, or images) in a human-subjects experiment; and (2) examined participants' decisions and opinion following the intervention. Specifically, we searched for the keywords *video, political, exposure, experiment, partisan, polarization, YouTube, and influence*, then filtered papers according to the criteria above. The resulting collection of studies is listed in this section.

A. List of Included Studies.

1. Hassin et al. (2007) (9)
2. Matz et al. (2017) (10)
3. Athey et al. (2023) (11)
4. Hameiri et al. (2014) (12)
5. DeMora et al. (2021) (13)
6. Sands (2017) (14)
7. Tappin et al. (2023) (15)
8. Mernyk et al. (2022) (16)
9. Petersen et al. (2021) (17)
10. Balieti et al. (2021) (18)
11. Guess et al. (2020) (19)
12. Wittenberg et al. (2021) (20)
13. Pink et al. (2021) (21)
14. Callaghan et al. (2021) (22)
15. Chu et al. (2021) (23)
16. Minozzi et al. (2015) (24)
17. Chang et al. (2019) (25)
18. Goldenberg et al. (2018) (26)
19. Asimovic et al. (2021) (27)
20. Enos (2014) (28)
21. Bail et al. (2018) (29)
22. Guess et al. (2021) (30)

B. Methods for Calculating Stimulus Exposure Time. For each study, we examined the stimuli used in the experiment and recorded the length of time for which subjects were exposed to the stimulus. This duration was sometimes directly reported in the methods or Supplementary Information; when the information was not reported, we identified the original stimuli whenever possible and computed the stimulus exposure time using conservative estimates:

1. In cases of **video stimuli**, e.g., Tappin et al. (2023) (15), we visited and manually compiled the lengths of each video stimulus.
2. In cases of **text-based stimuli,** we use a conservative estimate of 170 words per minute to compute the stimulus exposure time.
3. In cases of **social media-based stimuli**, e.g., Matz et al. (2017) (10), we used platform-specific data (e.g., from Facebook) to estimate values such as the average amount of time spent viewing social media advertising.
4. In cases of **in-person stimuli**, we used the reported length of time spent conducting in-person experiments (e.g., Sands (2017) (14)), divided by the estimated number of exposed subjects, to calculate the expected amount of exposure time.

Finally, in studies with multiple conditions, we averaged the estimated exposure time across each condition.

19. Prompts Used for GPT-4 and GPT-4V

A. First Impressions Baseline: Prompt Used for GPT-4V. You are an objective rater whose job is to judge the content of YouTube videos based on minimal information. You will be provided with the thumbnail of a YouTube video, the title of the video, the channel name, and the number of views. Your job is to estimate the content of the video as either 'supporting' or 'opposing' an increase to the minimum wage. Return 'pro' if you think the video supports an increase to the minimum wage and 'anti' if you think the video opposes an increase to the minimum wage. Even if it is unclear, please provide your BEST guess. You may NOT use the words 'sorry,' 'assist,' 'request,' or ANY OTHER ENGLISH WORDS in your response. You may ONLY use the words 'pro' or 'anti'. There are NO other options.

B. Continuous Measure of Extremity: Prompt Used for GPT-4V. You are an objective rater whose job is to judge the political leaning of YouTube videos. You will be provided with information, which may include: (1) the thumbnail of a YouTube video; (2) the origin channel of the video; and (3) the video transcript. Your job is to estimate the political stance of the video as accurately as you can, as a continuous score between -1 and +1. Positive scores mean a video is conservative, which means it is AGAINST minimum wage, and negative mean that a video is liberal, which means it is SUPPORTING the minimum wage. Moderate videos should be closer to zero (0). Use -1 and + 1 as the anchor points for the 'most extremely liberal' and 'most extremely conservative,' respectively. For example, if the video is conservative (i.e., AGAINST minimum wage), you should think of those that express stronger opposition to the minimum wage as more 'extreme.' You may look for signs of exaggerating the impacts of raising the minimium wage, or more intense language against the minimum wage. The more extreme the conservative view, the closer the rating to +1. Similarly, if the video is liberal (i.e., FOR minimum wage), you should think of videos that express stronger support for the minimum wage as more 'extreme.' You may look for signs of exaggerating the benefits and necessity of raising the minimum wage, as well as more intense language in support of the minimum wage. The more extreme the liberal view, the closer the rating to -1. In addition, if the video is from a more extreme partisan source, then it is more likely to be extreme, and you should update your judgements accordingly. Think of your role as quantifying the OBJECTIVE POLITICAL LEANING of political positions based on the information provided. If the leaning of a video is unclear, please provide your BEST guess. In cases where someone is being interviewed, please judge based on the leaning of the HOST, rather than the guest. You may NOT use the words 'sorry,' 'assist,' 'request,' or ANY OTHER ENGLISH WORDS in your response. You may ONLY use a number. There are NO other options. Information about the video is as follows:

C. Text Classification of Learning from Open Response: Prompt Used for GPT-4. You are an objective rater and helpful research assistant. Your goal is to analyze a response to a survey question about whether a participant learned anything after watching YouTube videos on the minimum wage. You are tasked with determining whether the respondent learned anything from the videos. Rate the responses on a binary scale, in which the score should be 1 if the respondent indicates that they learned something and 0 if they indicated they did not learn anything. You may NOT use ANY ENGLISH WORDS in your response, and your response MUST be an integer (0 or 1). If you are at all uncertain, please do your best. Here is one response:

References

1. K Arceneaux, M Johnson, *Changing Minds or Changing Channels?: Partisan News in an Age of Choice*, Chicago Studies in American Politics. (University of Chicago Press), (2013).
2. JN Druckman, MS Levendusky, What do we measure when we measure affective polarization? *Public Opin. Q.* **83**, 114–122 (2019).
3. W Lin, Agnostic notes on regression adjustments to experimental data: Reexamining freedman's critique. *Annals Appl. Stat.* **7**, 295–318 (2013).
4. RJ Simes, An improved bonferroni procedure for multiple tests of significance. *Biometrika* **73**, 751–754 (1986).
5. Y Benjamini, Y Hochberg, Controlling the false discovery rate: a practical and powerful approach to multiple testing. *J. Royal statistical society: series B (Methodological)* **57**, 289–300 (1995).
6. A Lai, et al., Estimating the ideology of political youtube videos. *Polit. Analysis* pp. 1–16 (2024).
7. H Hosseinmardi, et al., Causally estimating the effect of youtube's recommender system using counterfactual bots. *Proc. Natl. Acad. Sci.* **121** (2024).
8. H Hosseinmardi, et al., Examining the consumption of radical content on youtube. *Proc. Natl. Acad. Sci.* **118** (2021).
9. RR Hassin, MJ Ferguson, D Shidlovski, T Gross, Subliminal exposure to national flags affects political thought and behavior. *Proc. Natl. Acad. Sci.* **104**, 19757–19761 (2007).
10. SC Matz, M Kosinski, G Nave, DJ Stillwell, Psychological targeting as an effective approach to digital mass persuasion. *Proc. national academy sciences* **114**, 12714–12719 (2017).
11. S Athey, K Grabarz, M Luca, N Wernerfelt, Digital public health interventions at scale: The impact of social media advertising on beliefs and outcomes related to covid vaccines. *Proc. Natl. Acad. Sci.* **120**, e2208110120 (2023).
12. B Hameiri, R Porat, D Bar-Tal, A Bieler, E Halperin, Paradoxical thinking as a new avenue of intervention to promote peace. *Proc. Natl. Acad. Sci.* **111**, 10996–11001 (2014).
13. SL DeMora, JL Merolla, B Newman, EJ Zechmeister, Reducing mask resistance among white evangelical christians with value-consistent messages. *Proc. Natl. Acad. Sci.* **118**, e2101723118 (2021).
14. ML Sands, Exposure to inequality affects support for redistribution. *Proc. Natl. Acad. Sci.* **114**, 663–668 (2017).

15. BM Tappin, C Wittenberg, LB Hewitt, AJ Berinsky, DG Rand, Quantifying the potential persuasive returns to political microtargeting. *Proc. Natl. Acad. Sci.* **120**, e2216261120 (2023).
16. JS Mernyk, SL Pink, JN Druckman, R Willer, Correcting inaccurate metaperceptions reduces americans' support for partisan violence. *Proc. Natl. Acad. Sci.* **119**, e2116851119 (2022).
17. MB Petersen, A Bor, F Jørgensen, MF Lindholt, Transparent communication about negative features of covid-19 vaccines decreases acceptance but increases trust. *Proc. Natl. Acad. Sci.* **118**, e2024597118 (2021).
18. S Balietti, L Getoor, DG Goldstein, DJ Watts, Reducing opinion polarization: Effects of exposure to similar people with differing political views. *Proc. Natl. Acad. Sci.* **118**, e2112552118 (2021).
19. AM Guess, et al., A digital media literacy intervention increases discernment between mainstream and false news in the united states and india. *Proc. Natl. Acad. Sci.* **117**, 15536–15545 (2020).
20. C Wittenberg, BM Tappin, AJ Berinsky, DG Rand, The (minimal) persuasive advantage of political video over text. *Proc. Natl. Acad. Sci.* **118**, e2114388118 (2021).
21. SL Pink, J Chu, JN Druckman, DG Rand, R Willer, Elite party cues increase vaccination intentions among republicans. *Proc. Natl. Acad. Sci.* **118**, e2106559118 (2021).
22. B Callaghan, L Harouni, CH Dupree, MW Kraus, JA Richeson, Testing the efficacy of three informational interventions for reducing misperceptions of the black–white wealth gap. *Proc. Natl. Acad. Sci.* **118**, e2108875118 (2021).
23. J Chu, SL Pink, R Willer, Religious identity cues increase vaccination intentions and trust in medical experts among american christians. *Proc. Natl. Acad. Sci.* **118**, e2106481118 (2021).
24. W Minozzi, MA Neblo, KM Esterling, DM Lazer, Field experiment evidence of substantive, attributional, and behavioral persuasion by members of congress in online town halls. *Proc. Natl. Acad. Sci.* **112**, 3937–3942 (2015).
25. EH Chang, et al., The mixed effects of online diversity training. *Proc. Natl. Acad. Sci.* **116**, 7778–7783 (2019).
26. A Goldenberg, et al., Testing the impact and durability of a group malleability intervention in the context of the israeli–palestinian conflict. *Proc. national academy sciences* **115**, 696–701 (2018).
27. N Asimovic, J Nagler, R Bonneau, JA Tucker, Testing the effects of facebook usage in an ethnically polarized setting. *Proc. Natl. Acad. Sci.* **118**, e2022819118 (2021).
28. RD Enos, Causal effect of intergroup contact on exclusionary attitudes. *Proc. Natl. Acad. Sci.* **111**, 3699–3704 (2014).
29. CA Bail, et al., Exposure to opposing views on social media can increase political polarization. *Proc. Natl. Acad. Sci.* **115**, 9216–9221 (2018).
30. AM Guess, P Barberá, S Munzert, J Yang, The consequences of online partisan media. *Proc. Natl. Acad. Sci.* **118**, e2013464118 (2021).
